# Photorheological action plots

**DOI:** 10.1039/d6sc04925g

**Published:** 2026-08-10

**Authors:** Elizabeth Graham, Jan Hobich, Hansjörg Grützmacher, Kai Mundsinger, Josh Lipton-Duffin, Christopher Barner-Kowollik

**Affiliations:** a School of Chemistry and Physics, Centre for Materials Science, Queensland University of Technology (QUT) 2 George Street Brisbane QLD 4000 Australia christopher.barnerkowollik@qut.edu.au k.mundsinger@qut.edu.au; b Institute of Functional Interfaces (IFG), Karlsruhe Institute of Technology (KIT) Kaiserstraße 12 76131 Karlsruhe Germany christopher.barner-kowollik@kit.edu; c School of Chemistry, Sun Yat Sen University 510006 Guangzhou China

## Abstract

Wavelength-dependent photochemistry is often probed in solution *via* photochemical action plots using optical parametric oscillators (OPOs). These studies have shown that wavelength-dependent photochemical reactivity and absorption spectra are misaligned for a range of photochemical reactions. Crosslinked soft matter materials are often obtained *via* light-driven curing. Herein we introduce the concept of photorheological action plots for crosslinking reactions to assess their response to an identical number of photons at each wavelength. We employ the radical initiator aminobis(2,4,6-trimethylbenzoyl) phosphine oxide (BAPO-NH_2_) for photocuring in a polyethylene glycole diacrylate (PEG-DA) resin. We demonstrate that different evaluation methodologies of the time-dependent rheological measurements lead to similar photorheological action plots, showing a mismatch between the absorbance of the photoinitiator and the photorheological response. Critically, the photorheological action plot does not fully resemble the solution action plot of BAPO-NH_2_ initiated bulk methyl methacrylate (MMA) photopolymerization. Our study thus demonstrates that the most effective curing wavelength for cross-linking cannot (at least in the current case) be derived from the photoinitiator's absorbance spectrum nor a solution-based photochemical action plot.

## Abstract 2

The current study introduces photorheological action plots to map the wavelength-dependent change in mechanical properties of photoresins during curing.

## Graphical abstract

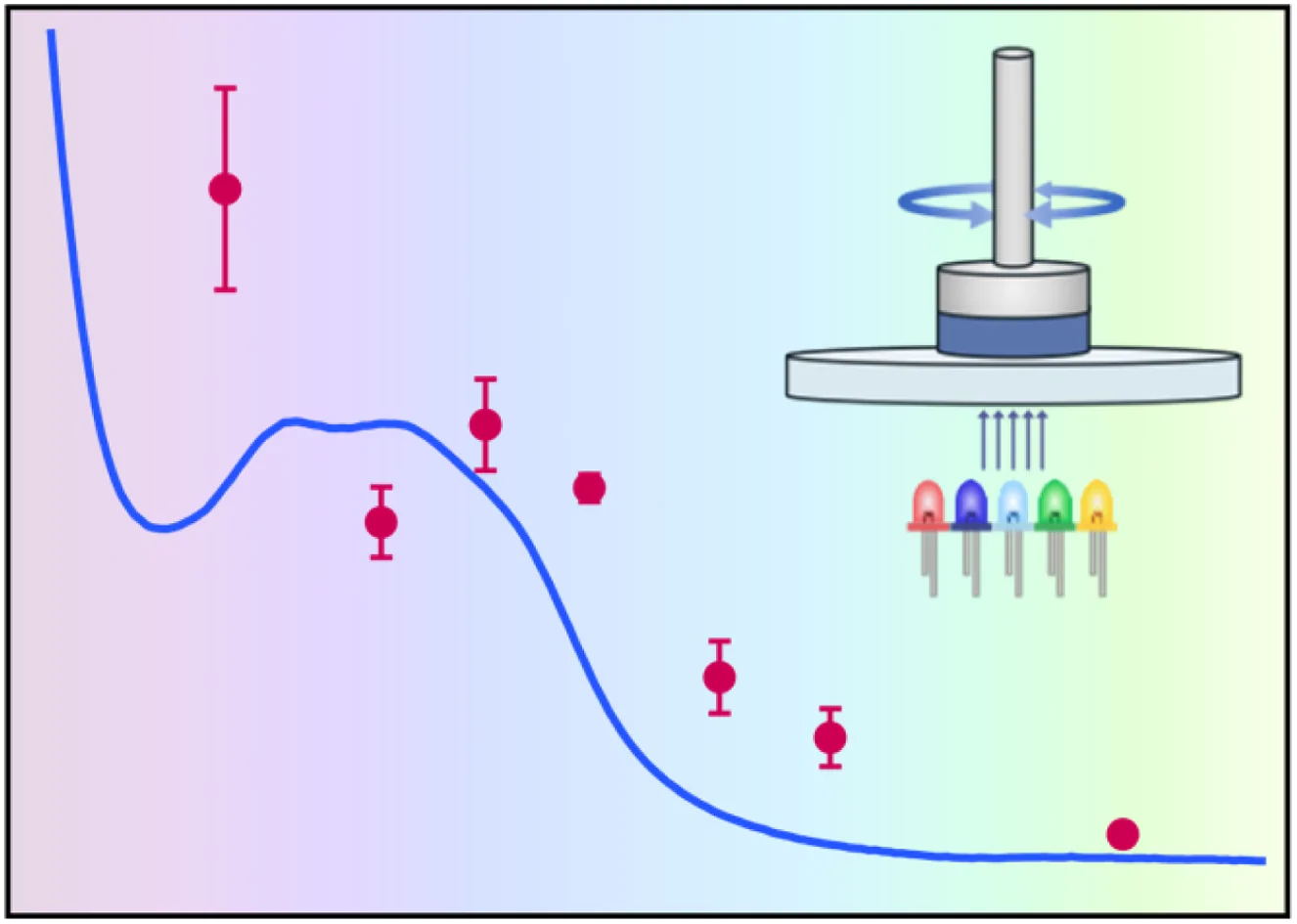

## Introduction

Applications targeting the light-based curing of photoresins have received substantial interest over the last decades and serve as an extremely versatile and effective toolbox for fields such as additive manufacturing,^1–4^ dental materials^5,6^ or adhesives.^7,8^ Specifically, a liquid photoresin is solidified by photopolymerization under irradiation with light. Volumetric 3D printing has emerged as a powerful new concept that is based on directly printing 3D structures within a vat filled with photoresin, offering the potential to drastically increase printing speeds.^9,10^ Furthermore, the scope of feasible designs is expanded as support structures for overhangs are no longer required, unlike in conventional layer-by-layer processes.^9^ Similarly, in the dental field the interest for high volume photocurable materials gained interest with the potential to avoid the tedious layer-by-layer curing processes and to ‘bulk-fill’ and solidify the material in a single step.^6^

Volumetric approaches notably require a high optical transparency at the employed printing wavelength to produce homogenous structures throughout the photoresin.^11^ The absorbance spectrum of the photoactive species (*i.e.* the photoinitiator) typically determines the optical transparency, and thus the intuitive approach of selecting the photoinitiator's absorbance maximum as the working wavelength is common. However, in 2017 the groups of Barner-Kowollik and Gescheidt^12^ observed a distinct red-shift of the maximum reactivity of a photoinitiator as compared to its absorbance maximum, a phenomenon that was subsequently observed repeatedly in a variety of photoreactions.^13–19^ Consequently, photoreactions can often be performed more efficiently at longer wavelengths with lower extinction coefficients and thus higher penetration depth. Systematic characterization of these wavelength-dependent reactivities is achieved *via* photochemical action plots,^11,20,21^ collected by repeatedly performing the reaction using an identical number of photons at selected monochromatic wavelengths. These experiments are typically performed in dilute model systems with low chromophore concentrations and are kept in the linear regime of the reaction kinetics (approx. below 30% conversion) to allow for a quantitative comparison of reaction yields obtained at each wavelength. Determination of reaction yields is often performed by either proton nuclear magnetic resonance (^1^H-NMR) spectroscopy, liquid chromatography-mass spectrometry (LC-MS), gravimetric analysis or more recently real-time UV-vis spectroscopy, providing quantitative insights into the wavelength-dependent reactivity of chromophores.^20,22^

The current state of the art for recording action plots is ideally suited for exploring photoreactivity in the solution state, such as *e.g.* photocycloadditions,^23^ photoswitching^24^ and low conversion photopolymerizations.^25^ However, transfer to applications in curing-based processes (*e.g.* for light-based additive manufacturing) brings challenges and demands for the development of alternative characterization approaches. Curing *via* polymer network formation proceeds from a liquid photoresin over a gel until a solid crosslinked material is obtained, making it challenging to track the conversion throughout the entire sample depth.^26^ Furthermore, for many curing-based applications the precise degree of conversion itself is secondary to whether the final material meets the required performance standards (*e.g.* mechanical properties), although in some contexts, such as biomedical applications, achieving sufficiently high conversion remains important to minimize the release of unreacted, potentially cytotoxic monomer.^27,28^ There is thus a need for action plot methodologies that investigate crosslinking systems, with the aim of characterizing the curing of photoresins with a focus on the material properties that are relevant to curing-based applications such as light-based additive manufacturing, dental materials or adhesives.

We herein introduce photorheological action plots *via* a custom-made photorheology setup for recording action plots (Fig. 1). The setup allows tracking the evolution of the loss modulus *G*′ as a measure of material stiffness, which affords quantitative insights into the progress of network formation in real time. It is important to note that the presented version of an action plot using a constant photoinitiator concentration at each wavelength captures the sum of all wavelength dependent effects. The conversion-dependent evolution of the photoresin entails the coupled photophysical, photochemical and optical effects.^29^ Consequently, *G*′ can be regarded as a parameter that reflects the overall reaction progress by encompassing the combined effects of numerous contributing factors. We thus introduce the first action plot of a polymer network formed inside a rheometer, employing an acrylate-based photoresin containing the photoinitiator aminobis(2,4,6-trimethylbenzoyl) phosphine oxide BAPO-NH_2_. Importantly, we highlight different approaches to evaluate the rheological data. Furthermore, a comparison to the solution-state photochemical action plot of BAPO-NH_2_ is discussed, highlighting differences of the respective techniques.

**Fig. 1:**
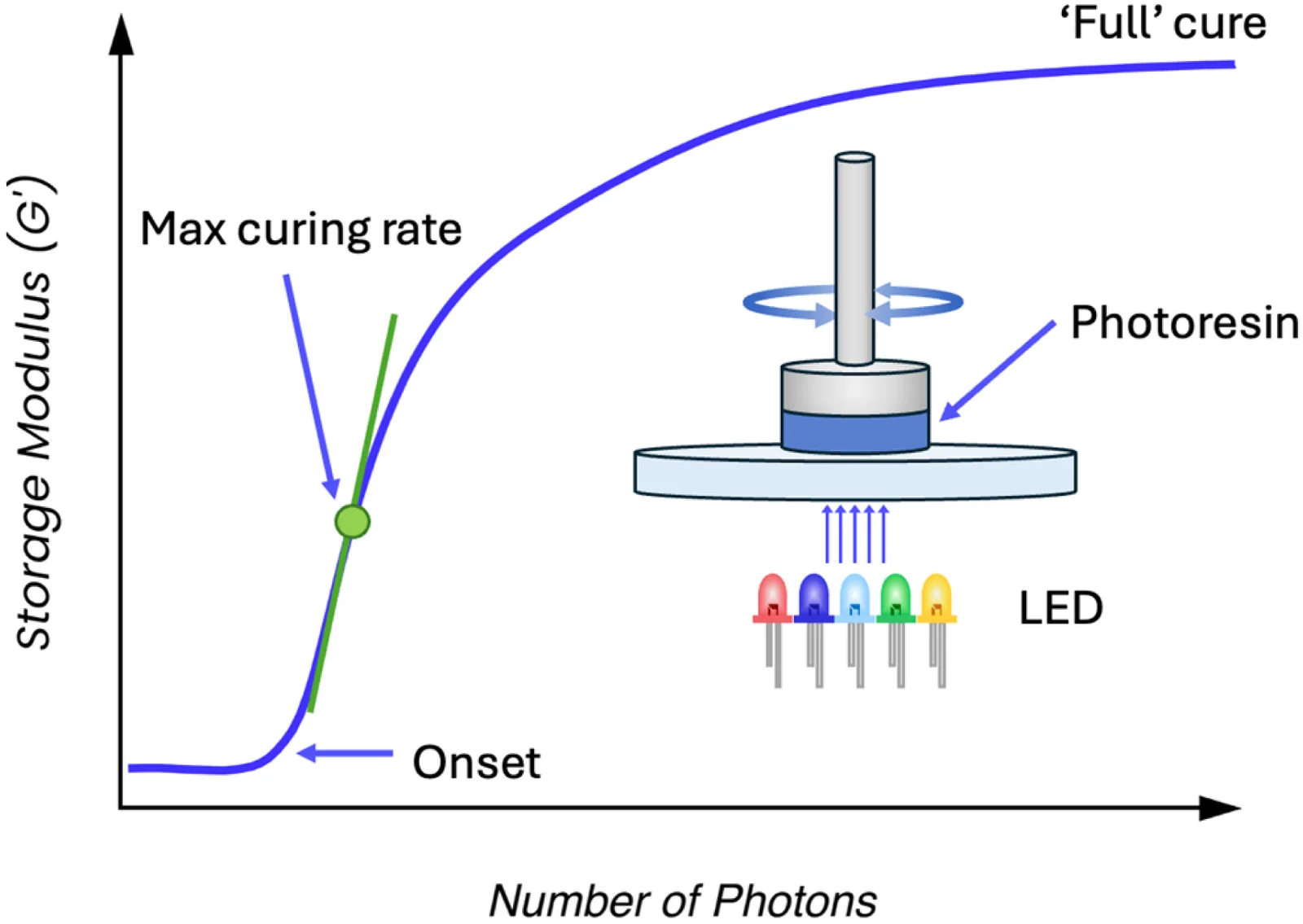
Overview of the photorheological approach used to construct wavelength-dependent action plots. A rotational rheometer was used to measure the evolution of storage modulus as a function of photon dose as the sample was cured under irradiation with different wavelengths. A schematic change of the storage modulus (*G*′) as a function of photon dose is shown, illustrating onset of curing and maximum curing rate.

## Results and discussion

Since the aim of the current study is the development of an action plot methodology based on mechanical property characterization of a polymerizing system rather than *via* spectroscopic or gravimetric method, a custom apparatus was built in the rheometer to supply photons to a photoresin *in situ* while the evolution in the sample's mechanical properties were measured. The properties measured here were the storage modulus (*G*′) and loss modulus (*G*″), and several methods to evaluate the data were assessed. The resulting conversion data are compared with the gravimetric action plot data for a BAPO derivative that has shown a strong red-shift in its solution-state action plot.

### Preparation of the photoresin

The photoinitiator is the key factor for reaction efficiency and choice of curing wavelength. Among commercially available photoinitiators, bisacylphosphine oxides are widely used due to their high efficiency and biocompatibility.^30,31^ They are employed across a broad range of industrial and biomedical applications, including stereolithographic fabrication of tissue scaffolds,^32–34^ light-curing dental resins and adhesives,^35,36^ 3D printed hydrogels,^37^ biosensor nanostructures^38^ and catalytic metal-polymer nanocomposites.^39^ A specific compound in the family of bisacylphosphine oxides, namely BAPO-NH_2_, was recently examined by Thijssen *et al.*,^11^ showing a pronounced red-shift of close to 50 nm between reactivity and absorbance maxima in a solution photopolymerization action plot study, enabling polymerization at wavelengths associated with comparatively low absorption at the red side of the absorption spectrum. Photopolymerization of methyl methacrylate (MMA) with BAPO-NH_2_ at low monomer conversion serves as model system for action plot studies, yielding important insights into the general wavelength-dependent reactivity of the photopolymerization process. However, different approaches must be developed when targeting applications based on polymer network formation, as characterization techniques such as gravimetry, NMR spectroscopy or LC-MS are challenging to employ for quantitatively tracking conversion in crosslinked systems.

Photoresins for network formation are often based on photopolymerization of (meth)acrylates.^40^ The basic components include a monofunctional monomer, a crosslinker with at least two (meth)acrylate moieties for network formation and a photoinitiator to trigger the photopolymerization. Solvents may also be added to improve consistency, homogeneity and handling of the liquid photoresin for the photorheology experiments.^40^ A model photoresin was used in these experiments to ensure that the reaction kinetics and sample handling were suitable for testing the photorheology concept. Using a model photoresin minimized the number of variables introduced into the experiment while allowing optimization of the test conditions. The model photoresin used for these photo-rheological measurements was selected according to several practical and optical requirements. The photoresin needed to be sufficiently transparent to minimize scattering during irradiation, ensuring that the incident wavelength penetrated the full sample thickness without introducing spatial gradients during curing. In addition to optical clarity, the formulation required a balance of viscosity and reactivity that enabled uniform loading between the rheometer plates, cure times ranging from no less than 10 minutes to no longer than two hours and minimal adhesion to the rheometer plates after gelation.

The transmittance of light through the formulation was calculated from UV-vis absorbance data over the 300–500 nm range (refer to SI Fig. S7). These indicate that transmittance exceeds 95% for wavelengths above 340 nm for sample thicknesses between 0.05 mm and 0.8 mm, with increased attenuation only occurring at the shortest wavelengths, confirming that the chosen formulation and sample geometry allowed for an accurate comparison of wavelength-dependent curing behavior across the selected wavelength region from 360 to 500 nm. The final sample thickness of 0.2 mm was chosen to maximize light transmission, while maintaining reliable mechanical contact between the rheometer plates.

The employed photoresin as shown in Fig. 2 consisted of dimethyl acrylamide (DMA, 5 vol% 0.08 M), polyethylene glycol diacrylate, average *M*_n_ = 700 g mol^−1^ (PEG-DA 700, 5 vol% 0.486 M), dimethyl sulfoxide (DMSO, 40 vol% 5.63 M) and polyethylene glycol, average *M*_n_ = 400 g mol^−1^ (PEG 400, 50 vol% 1.4 M) and BAPO-NH_2_ (1 mM). This combination provided ideal viscosity, appropriate gelation behavior and effective light transmission through the sample. All components of the mixture were used as received and without further preparation (*e.g.* degassing) to mimic the conditions likely to be encountered in a commercial additive manufacturing setting. Further, the rheological experimental conditions did not allow for prevention of oxygen incursion during sample preparation and testing.

**Fig. 2:**
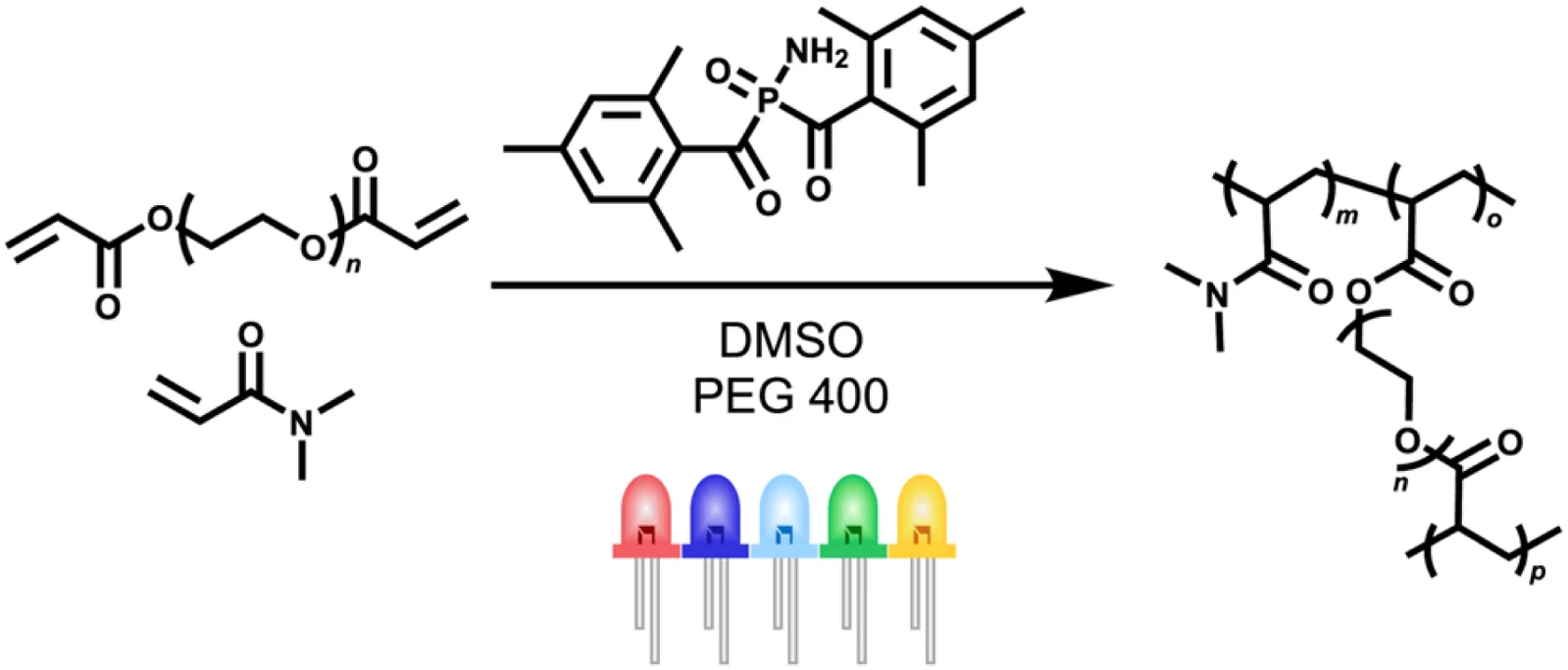
Light-induced polymer network formation of the photoresin based on DMA, PEG DA 700, DMSO, PEG 400 and BAPO-NH_2_ as photoinitiator.

### Rheological action plots

Rheology was selected to characterize the progress of a growing polymer network from liquid to a solid state as it enables real-time measurement of the evolving viscoelastic properties. The storage modulus or elastic modulus (*G*′), represents the elastic component of a viscoelastic material and quantifies the amount of energy stored during deformation. The typical evolution of *G*′ in rheology involves a frequency-dependent or time-dependent increase, often signifying the transition from a liquid-to a solid-like structure due to network formation, molecular entanglement and gelation.^41^ As a sample undergoes curing, the *G*′ evolution typically follows a three stage profile. Before the start of the curing process, the sample exhibits a low *G*′, often exceeded by its loss modulus (*G*″). Throughout light exposure *G*′ increases, eventually reaching a plateau, indicating that the sample is fully cured. The results are typically presented on log-linear or log-log axes to allow for a better visualization of the data as the *G*′ changes across several orders of magnitude.^42^

Herein, a dedicated photorheological apparatus was developed to measure changes in *G*′ under LED irradiation at discrete wavelengths (refer to SI Section 4.1 for a detailed schematic drawing of the set-up and light delivery system and the emission spectra of the employed LEDs in Fig. S1). It is critical to note that our setup allows for delivery of an identical photon number at each employed wavelength, which is a prerequisite for a photochemical action plot. The storage modulus of crosslinking systems scales linearly with degree of cure under appropriate conditions,^43^ allowing *G*′ to serve as a quantitative proxy for conversion throughout the photochemical action plot.

For the photorheological action plot measurement, the evolution of *G*′ was tracked as a function of the number of incident photons (*N*) for different wavelengths ranging from 360 to 500 nm (Fig. 3). All curves follow the same general trend and initially exhibit constant *G*′ until a critical *N* is reached (induction), marking the onset of a sharp increase which then progressively slows (curing), approaching a plateau (full cure) at approximately 3.8 kPa. When irradiated at the shortest wavelength (*i.e. λ*_max_ = 360 nm), *G*′ increased rapidly after a short induction period and the sample cured fully with exposure to the fewest number of photons across all the experiments performed. At the wavelengths from *λ*_max_ = 384 to 453 nm, the onset and rate of development of measurable modulus growth was variable and the magnitude of the curing response was similar across this range. At the longest wavelengths examined (*i.e. λ*_max_ = 498 nm), the *G*′ response was slow to initiate and develop with an onset after a dose of approximately 2.5 × 10^19^ photons, while the onsets for shorter wavelengths were in the range of 5 × 10^17^ to 1 × 10^18^ photons. Furthermore, the *G*′ plateau was not fully reached at *λ*_max_ = 498 nm, indicating that the sample did not fully cure within the experimental photon dose.

**Fig. 3:**
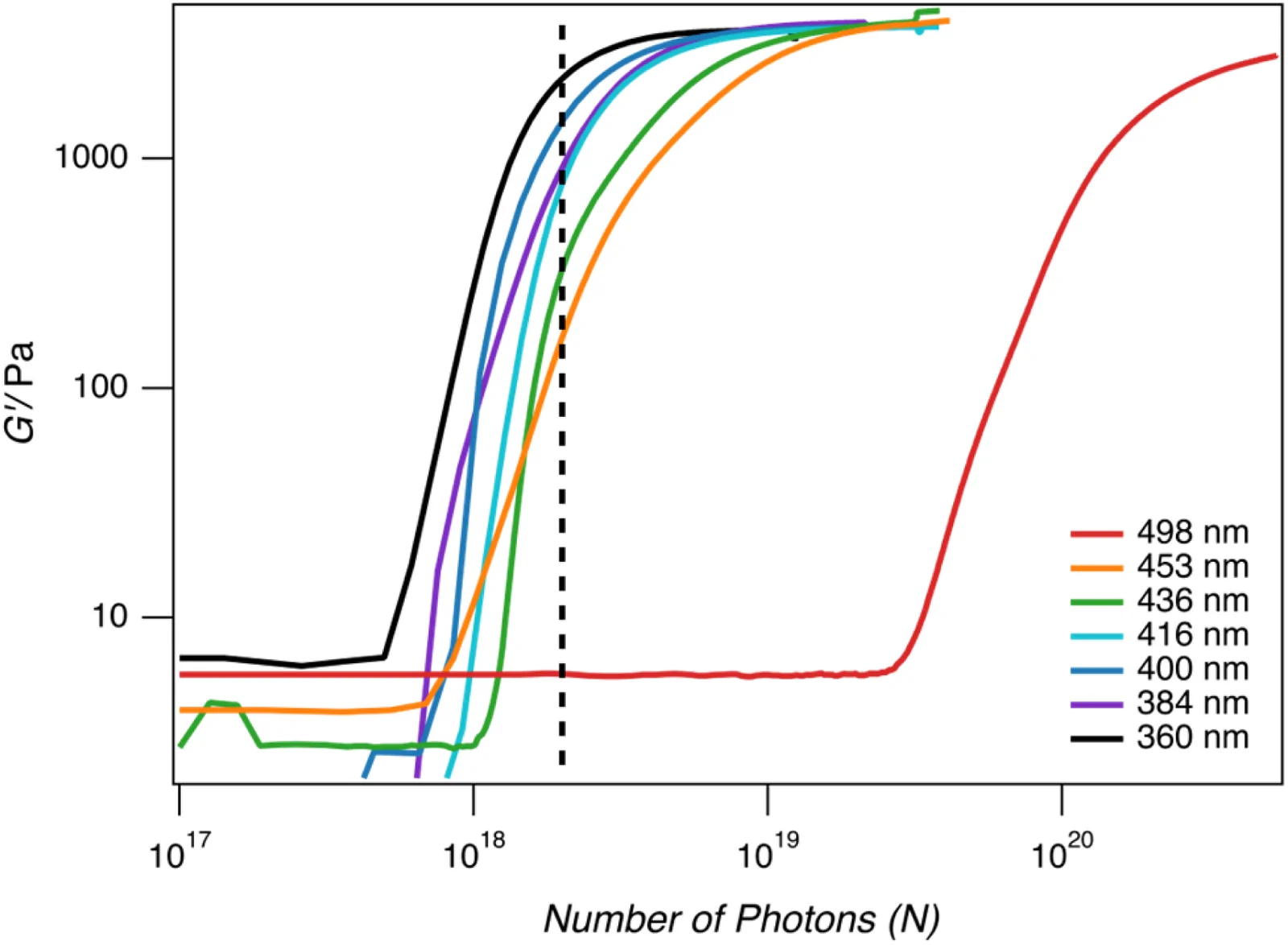
Evolution of storage modulus (*G*′) for the photoresin as a function of photon number at different wavelengths. The storage modulus evolution is shown for LEDs emitting at *λ*_max_ = 360, 384, 400, 416, 436, 453, and 498 nm, with photon number calibrated irradiance measurements. Each curve represents the averaged response of triplicate experiments. The shift in onset, photon number and rate of modulus development across wavelengths reflects wavelength-dependent photoreactivity under otherwise identical curing conditions. The dashed line represents the curing state evaluated herein at a number of photons of *N* = 2 × 10^18^.

### Analytical approaches for photorheological action plots

Action plots are usually performed by exposing a dilute solution of a chromophore to a nominally fixed number of photons and measuring the conversion of the starting materials at different wavelengths. An appropriate number of photons is selected to remain within the linear range of kinetics (typically below 30% conversion), allowing for the quantitative comparison of the separate data points. The curing progress of a photoresin is more challenging to assess, since the sample changes from a liquid to a gel or solid state. Furthermore, with additive manufacturing applications in mind, the important parameter is not the degree of conversion of monomer units, but rather the resulting material properties, such as *G*′.

A further advantage of real time tracking of *G*′ is that a continuous dataset is produced, as opposed to the single data point produced by solution methods. The continuous dataset allows for different approaches to constructing an action plot and the data analysis method can be driven by the application requirements. For instance, the most important parameter for a given material might be the onset of curing, where the mechanical properties start to change significantly, providing insights into the threshold where solidification of the resin occurs in 3D printing. Wavelength-orthogonal fabrication of different materials could thus be simplified by exploiting wavelength dependent degrees of curing onset. Conversely, the stage of curing at a selected number of photons, like the approach for a solution action plot, can be of interest. Another potential approach is to compare the maximum rate of change of *G*′ (d*G*′/d*N*_max_) across different wavelengths, providing a photon dose independent metric for curing kinetics. The number of photons required to reach d*G*′/d*N*_max_ was also considered. We evaluated the data according to the four different methods mentioned above, however, we will focus here on the fixed-dose approach as an analogue to the solution action plot and the maximum rate of change as a more holistic and dose-independent metric. A comparison with the action plots based on the onset and number of photons to reach d*G*′/d*N*_max_ can be found in SI Section 5.

Initially, a fixed number of photons analysis approach was applied to the rheological dataset, analogous to the approach used in classical action plots, with the difference that the fixed number of photons was chosen to intersect as many *G*′–*N* curves as possible at the lowest possible photon numbers. The intersection of the selected *N* = 2 × 10^18^ with all the *G*′–*N* curves is shown in Fig. 3. Comparison of the *G*′ values at different wavelengths for *N* = 2 × 10^18^ enabled the construction of a fixed photon number action plot, shown in Fig. 4. The local maximum of the reactivity profile at 400 nm appears red-shifted by approximately 15 nm from the first local absorbance maximum at 385 nm, reflecting a more efficient curing process at longer wavelengths than expected from the absorbance spectrum alone.

**Fig. 4:**
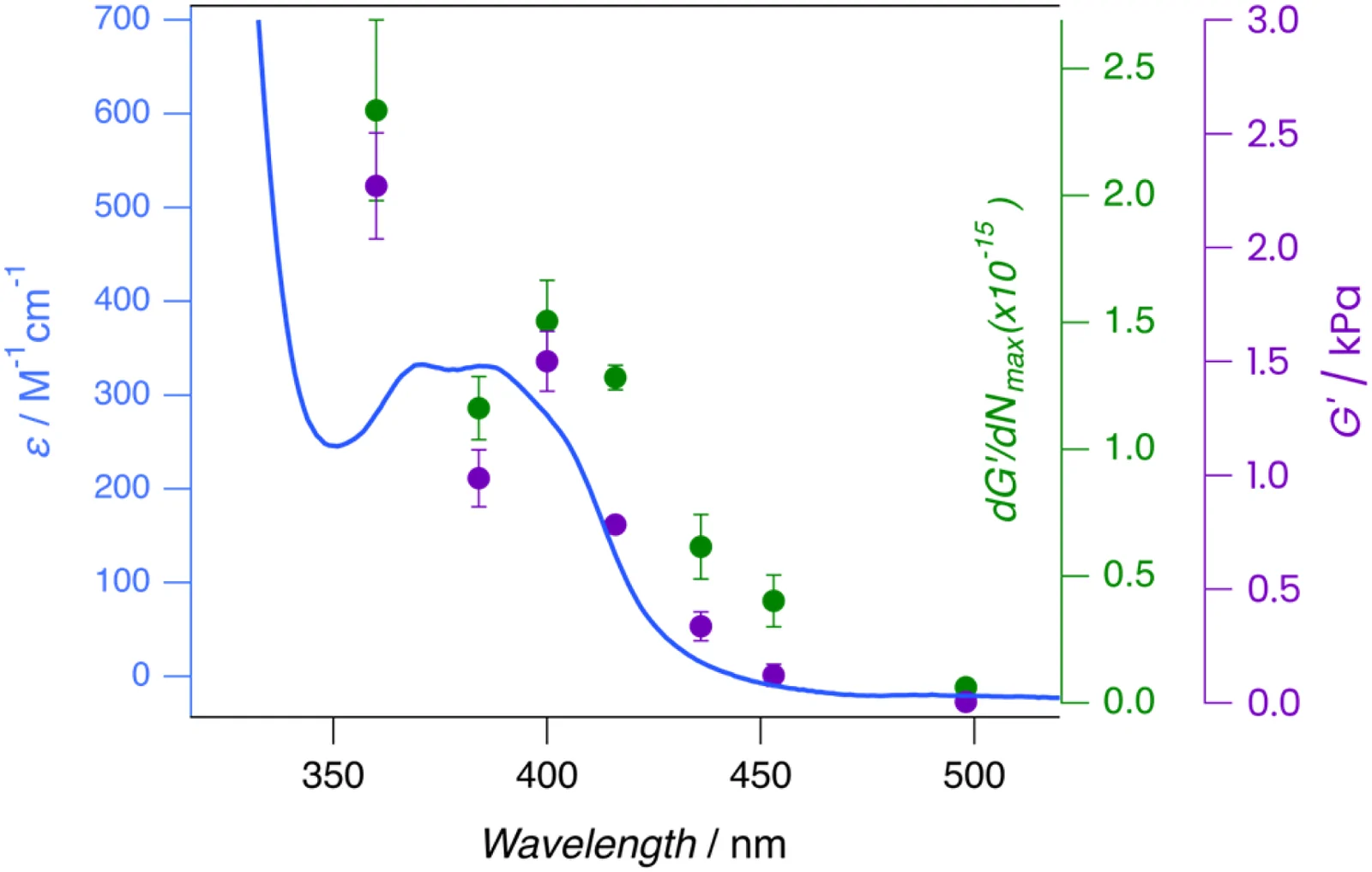
Comparison of two different approaches to construct an action plot: fixed number of photon rheological action plot depicting *G*′ for different wavelengths at a photon number of 2 × 10^18^ (purple) and d*G*′/d*N*_max_ rheological action plot depicting the maximum rates of change of *G*′ for different wavelengths (green). Both interpretations suggest a red shift in the reactivity profile of close to 15 nm with respect to the absorption profile (blue line).

The fixed number of photon representation is limited in the sense that it only represents the different *G*′ values at a specific number of photons compared across different wavelengths. Thus, the above analysis method gives detailed insights only for very specific irradiation conditions. Furthermore, the *G*′ curves cannot be compared in a linear regime as is the case for a solution action plot, thus the choice of *N* is somewhat subjective and may have a significant impact on the resulting action plot. Processes that evolve on different timescales and/or with different photon doses cannot be fully captured using the above approach. For instance, the fixed-*N* action plot in Fig. 4 suggests that no curing should occur at an irradiation wavelength of *λ*_max_ = 498 nm despite measurable curing occurring at larger exposures (*N* > 2.5 × 10^19^), leading to a general underestimation of extended induction periods and slower modulus growth, especially at longer wavelengths with extinction coefficients close to zero.

An alternative approach for the evaluation of the *G*′ curves to construct an action plot is to compare the maximum rates of curing (d*G*′/d*N*_max_), determined by differentiating *G*′ with respect to photon dose and identifying the peak value of the differentiated curve. The differentiated curves are shown in SI Fig. S14b. The resulting action plot, shown in Fig. 4, also shows a close to 15 nm red shift of the reactivity profile maximum from the first local maximum of the absorbance spectrum at 385 nm. The curing efficiency interpreted from the d*G*′/d*N*_max_ is higher for longer wavelengths *λ*_max_ = 436 nm and *λ*_max_ = 453 nm compared to the fixed-dose action plot, which might be due to the selection of *N* for the latter. Furthermore, the delayed curing at a wavelength of *λ*_max_ = 498 nm is represented in the d*G*′/d*N*_max_ action plot, while it appears to be zero for the fixed-dose action plot. Overall, the metric of maximum rate of change is clearly defined and independent of an arbitrary, subjective choice of *N* and robustly captures the sum of all effects of the curing process, reliably reflecting the progressing network formation. Accordingly, the maximum rate metric was selected as the most general and holistic photorheological analogue to a solution action plot. However, when the targeted number of photons is known, the fixed-dose approach may be preferable, as it proceeds under defined conditions. For example, when *G*′ at the curing threshold is known, it would be possible to identify which wavelengths could be employed in an orthogonal manner *e.g.* for multi-material 3D printing.^44,45^

### Wavelength-dependent activity comparison between photopolymerization and photo-induced crosslinking

The selection of BAPO-NH_2_ as the photoinitiator for establishing the photorheological action plot was driven by a previously reported, markedly red-shifted action plot obtained under solution conditions examining the photopolymerization of methyl methacrylate (MMA).^11^ Consequently, this dilute solution action plot serves as a useful benchmark for comparison with the photorheological approach. Comparison of the d*G*′/d*N*_max_ rheological action plot with the solution polymerization action plot (Fig. 5) illustrates that both show a red shift of the reactivity maximum compared to the local absorbance maximum at 385 nm. The red shift in both fixed photon number and d*G*′/d*N*_max_ rheological action plots is close to 15 nm to a single local maximum. For the solution action plot two maxima with similar degrees of conversion (*i.e.* 12.5% and 12.0%) are observed at 420 and 460 nm with respective red shifts of close to 50 and 80 nm. In the rheological action plot, a red shift is still evident, yet the displacement of the activity maximum is less pronounced, and the wavelength-dependent reactivity more closely follows the general trend of the UV-vis spectrum. However, even though a second action plot maximum is not observed for the rheological measurements, the reactivity extends well beyond the primary absorption band for both the d*G*′/d*N*_max_ and solution action plots, evidencing significant long wavelength reactivity (*i.e.* at 500 and 510 nm, respectively) at low extinction coefficients.

**Fig. 5:**
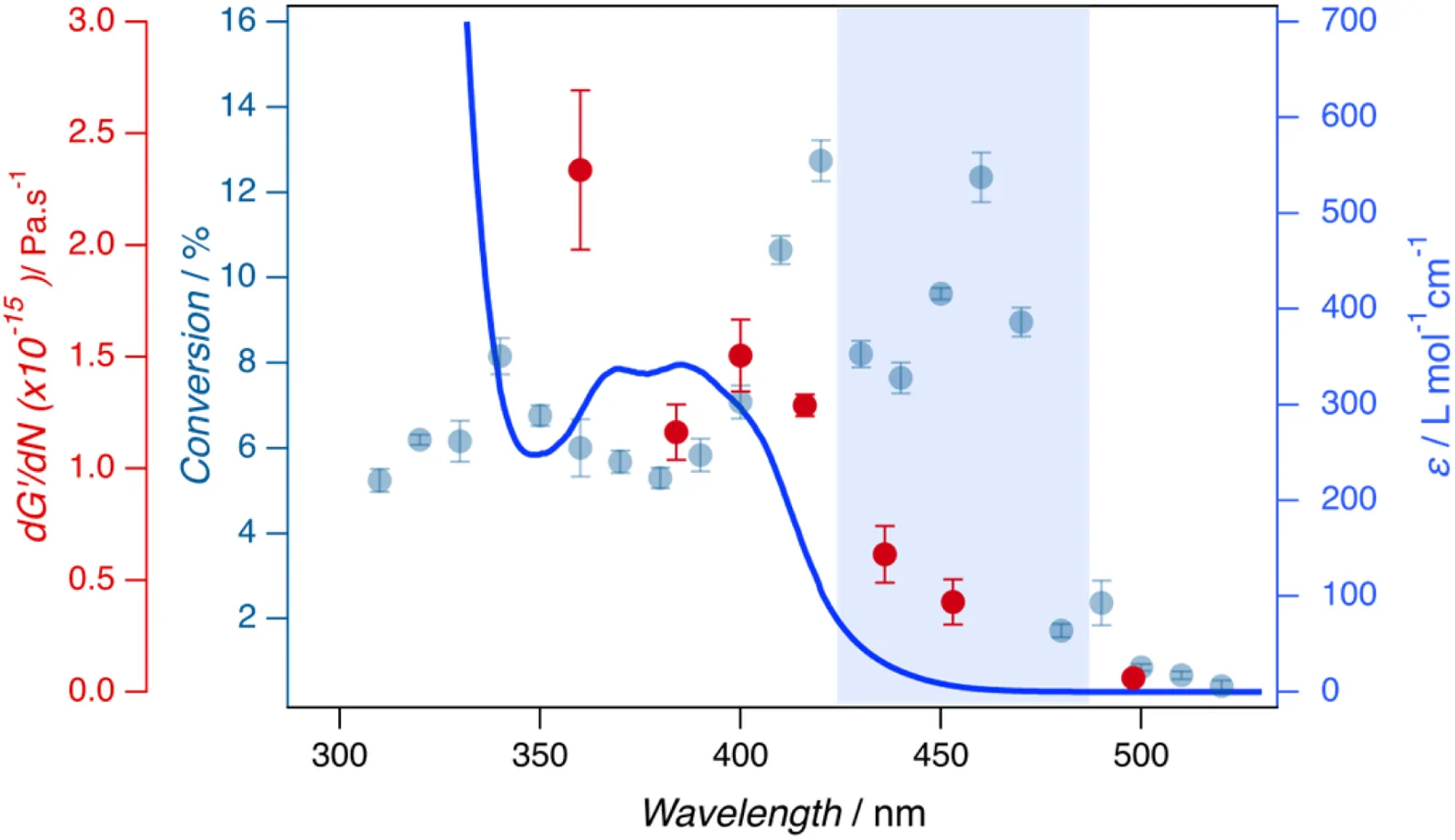
Absorbance spectrum of BAPO-NH_2_ in bulk MMA (blue line), overlaid with the herein established rheological (red) and the earlier recorded solution-based (blue) action plots for comparison. In both cases the action plot maxima appear red-shifted compared to the absorbance band, with differences especially in the shaded region between 420 and 490 nm. The solution-based action plot data was adapted and reproduced from ref. 13.

Notwithstanding the fact that both action plots are recorded at constant initiator concentration and capture photophysical, photochemical and optical effects, there are several factors that may explain the differences in the solution based and rheological based action plots. First, the actual reaction type and conditions are very different for both approaches. The solution setup is based on a bulk photopolymerization of MMA, yielding linear polymer chains and the conversion of the monomer is measured gravimetrically. In contrast, the reaction for photorheological action plots consists of multiple components in addition to photoinitiator and monomer, (*i.e.* crosslinker and solvent), and photopolymerization leads to the formation of an interconnected polymer network. While the conversion is kept below 15% to stay within the linear range of kinetics for solution action plots, the rheological measurements continue until the sample is fully cured and *G*′ reaches a plateau, thus the rheological action plot depends on non-linear kinetics and a continuous increase in viscosity. Furthermore, the laser employed in solution action plot measurements can deliver monochromatic light at each wavelength compared with the narrow-disperse LEDs of the photorheology setup. Thus, the fact that LEDs were employed could lead to a considerable loss of structure, as is evident when comparing action plots obtained with laser or LED irradiation for Ivocerin as photoinitiator in bulk MMA polymerization.^46,47^ Finally, formulations containing crosslinkers will show an increased degree of scattering, making comparisons with non-scattering systems challenging. In general, the solution action plot is based on an optimized model system that gives key insights into the sum of all wavelength-dependent effects and conversion of chromophores. The photorheological action plot is application oriented and enables the direct characterization of the curing of a photoresin by measuring the actual change in materials properties which might be especially interesting for the field of 3D printing.

Although photorheology provides the ability to understand wavelength-dependent curing behavior, it also has practical limitations. First and foremost, characterization is only possible for curing systems that drastically change their materials properties as a function of photon number such as photopolymerization. Moreover, the method requires sufficient optical transparency for uniform sample irradiation; strongly absorbing or scattering systems may display additional wavelength-dependent effects. The above noted scattering was also examined as a potential source of discrepancy in the current study but was shown to be minimal for the current system (refer to SI Fig. S8). Furthermore, the mechanical shearing inherent to rheology may facilitate radical transport and chain mobility, thereby potentially accelerating and distorting the reaction kinetics compared to a stationary photoresin. Sample compatibility with the rheometer is also essential: adhesive systems may bond irreversibly to the plates unless disposable components are used and volatile formulations may change composition during testing. The technique is best suited to materials with moderate to high viscosity that can be loaded into a parallel-plate geometry. Low-viscosity samples typically require cup-and-bob or double-gap geometries, which do not accommodate controlled irradiation, while cone-and-plate geometries introduce slightly varying thicknesses that complicate light-transport analysis.

Photorheology inherently mimics the mechanical environment of processing operations such as additive manufacturing, coatings and surface curing, and within the above-described constraints, the method offers unique insights into curing processes that cannot be captured by fixed-dose or purely spectroscopic approaches.

## Conclusions

We demonstrate that photorheology provides a powerful and complementary approach to classic gravimetric and spectroscopic methods for understanding and mapping wavelength-dependent photochemical effects in an action plot. By monitoring the evolution of storage modulus, *G*′, under LED irradiation at different wavelengths in a custom-built photorheometer, the combined influences of light absorption, radical generation and network formation are captured within a single experiment. This holistic view offers particular value in curing systems where mechanical development governs functional performance *e.g.* in 3D printing.

The proof-of-concept for the photorheological action plot was performed by characterizing the curing of an acrylate-based photoresin with photoinitiator BAPO-NH_2_. Different approaches to construct an action plot from the *G*′ curves were discussed with a focus on the comparison of *G*′ after the delivery of a fixed number of photons and the comparisons of the maximum rate of change of *G*′. Among the analytical metrics, the maximum rate of change of *G*′ curing provided the most reliable and mechanistically meaningful representation of wavelength-dependent behavior, capturing reactivity across the full spectral range more faithfully than other approaches. A red shift of close to 15 nm was observed for the first local action plot maximum of BAPO-NH_2_ to the local absorbance maximum and ongoing reactivity until close to 500 nm. Similar red-shifted reactivity was observed for the same photoinitiator in a previously reported solution action plot, underlining the utility of the photorheological approach as a complementary technique for understanding wavelength-dependency of curing systems.

The photorheological approach has practical constraints, including requirements for sample transparency, viscosity and compatibility with parallel plate geometries. Nevertheless, it offers unique insight into photocuring processes that are difficult to probe by other means and is particularly relevant for materials used in additive manufacturing, coatings and applications where mechanical development is directly tied to performance.

Overall, we herein establish photorheology as a practical and sensitive method for probing wavelength-dependent curing behavior, especially in systems where mechanical evolution accompanies photochemical conversion. Future studies will extend our approach to additional photoinitiators and material systems to further explore red-shift phenomena and develop mechanistically grounded action-plot methodologies that more accurately reflect curing behavior in real formulations. Further, we aim to couple a photorheometer with optical parametric oscillators for monochromatic wavelength precision.

## Author contributions

L. G. conceptualization, experiments, data analysis, original manuscript drafting; J. H. data analysis, manuscript drafting; H. G. development of BAPO-NH_2_ synthesis, manuscript drafting; K. M. conceptualization, data analysis, manuscript drafting, supervision; J. L. -D. conceptualization, data analysis, manuscript drafting, supervision; C. B. -K conceptualization, data analysis, manuscript drafting, supervision, funding acquisition.

## Conflicts of interest

There are no conflicts to declare.

## Supplementary Material

SC-017-D6SC04925G-s001

## Data Availability

All data, *e.g.* synthetic procedures, characterization data, photophysical data and a detailed description of the photorheological action plot setup are available in the supplementary information (SI) provided alongside this article. Supplementary information is available. See DOI: https://doi.org/10.1039/d6sc04925g.
