# Supplementary material for: Photorheological action plots
Source: Chem Sci. 2026 Aug 10;17(39):18658–64. doi: 10.1039/d6sc04925g (PMC13479091; doi:10.1039/d6sc04925g)
Supplement: Supplementary file 1 [file SC-017-D6SC04925G-s001.pdf]

Supplementary Information (SI) for Chemical Science.
This journal is © The Royal Society of Chemistry 2026

Supplementary Information

Photorheological Action Plots

Elizabeth Graham,[1] Jan Hobich,[2] Hansjörg Grützmacher,[3] Kai Mundsinger,[1] Josh Lipton-Duffin,[1] Christopher Barner-Kowollik[1,2]**

[1] School of Chemistry and Physics, Centre for Materials Science, Queensland University of Technology (QUT), 2 George Street, Brisbane, QLD 4000, Australia.

[2] Institute of Functional Interfaces (IFG), Karlsruhe Institute of Technology (KIT), Kaiserstraße 12, 76131 Karlsruhe, Germany.

[3] School of Chemistry, Sun Yat Sen University, 510006 Guangzhou, China

E-mail:

christopher.barnerkowollik@qut.edu.au, christopher.barner-kowollik@kit.edu,
k.mundsinger@qut.edu.au

Contents

1 Materials and Methods 3

1.1 Materials 3

1.2 Nuclear Magnetic Resonance (NMR) Spectroscopy 3

1.3 Ultraviolet-Visible (UV-Vis) Spectroscopy 3

1.4 Light Emitting Diode (LED) Characterization 3

2 BAPO-NH_2 - Synthesis and Characterization 5

2.1 Synthesis 5

2.2 Characterization via NMR 5

3 Preparation of the Photoresin 7

3.1 Preparation 7

3.2 UV-Vis Absorbance of the Photoresin 8

3.3 Transmittance of the Photoresin 9

3.4 Light Absorbance in the Photoresin 10

4 Photorheological Measurements 12

4.1 Photorheometer Setup 12

4.2 Photorheological Experiments 17

5 Construction of Photorheological Action Plots for BAPO-NH_2 18

5.1 Different Types of Photorheological Action Plots 18

5.2 Alternative Methods for Photorheology Action Plot Construction 19

5.3 Visualization of Photorheological Action Plot Evaluation Methods 20

6 References 22

1 Materials and Methods

1.1 Materials

The following chemicals were used as received without further purification: Dimethyl acrylamide (DMA, 99%, Merck), dimethyl sulfoxide (99.9%, Univar), polyethylene glycol 400 (PEG 400, Merck), polyethylene glycol diacrylate 700 (PEG-DA 700, average M_n = 700, 50 - 150 ppm MEHQ as inhibitor, 200 - 400 ppm BHT as inhibitor, Merck), methyl methacrylate (MMA, ≥ 99%, ≤ 30 ppm MEHQ as inhibitor, Merck), phenyl-bis(2,4,6-trimethylbenzoyl)phosphinoxide (BAPO, 97%, Merck).

1.2 Nuclear Magnetic Resonance (NMR) Spectroscopy

^{1}H, ^{13}C and ^{31}P-NMR spectra of the compounds were recorded on a *Bruker* Neo 400, equipped with a 5 mm BBO-Probe (5 mm), (^{1}H: 399.72 MHz, ^{13}C: 100.51 MHz, ^{31}P: 161.81 MHz). The δ-scale was normalized relative to the magnetic resonance arising from the residual solvent signal of $CHCl_3$ ^{1}H spectra and for ^{13}C spectra on the middle signal of the $CHCl_3$ triplet, respectively. The multiplicities were reported using the following abbreviations: s for singlet, d for doublet, t for triplet, q for quartet, p for pentet, m for multiplet and br for broad signal.

1.3 Ultraviolet-Visible (UV-Vis) Spectroscopy

UV-Vis spectra were recorded on a *Shimadzu* UV-2700 spectrophotometer equipped with a CPS-100 electronic temperature control cell positioner. Samples were measured in *Hellma Analytics* quartz high precision cells with a path length of 10 mm at 25 °C.

1.4 Light Emitting Diode (LED) Characterization

The LEDs were employed at a photon flux of $5 \cdot 10^{15}$ s^{-1} for their characterization and all photorheology experiments. LED output energies were recorded using a *Thorlabs* S401C thermopile sensor, with an active area of 100 mm^2 and a wavelength range of 190 nm - 20 µm, connected to a *Thorlabs* PM400 energy meter console. The emitted power from each LED was measured for 60 s at the same distance from the sensor as in the photoreaction from the center of the crimp vial, after which the mean and standard deviation of the emitted wavelengths were determined. LEDs were mounted on a heatsink and fan cooled with ambient air during measurement to minimize any thermal effects on the emission power or sensor performance.

LED emission spectra (**Figure S1**) were recorded using an *Ocean Optics* FLAME-T-UV-VIS spectrometer, with an active range of 200-850 nm, an integration time of 300 ms and averaging over 10 scans. Emission profiles were fitted in IgorPro 8 using a linear regression method with an exponentially modified Gaussian model function, and the wavelength at the maximum emission was used as the wavelength for each photorheological experiment.

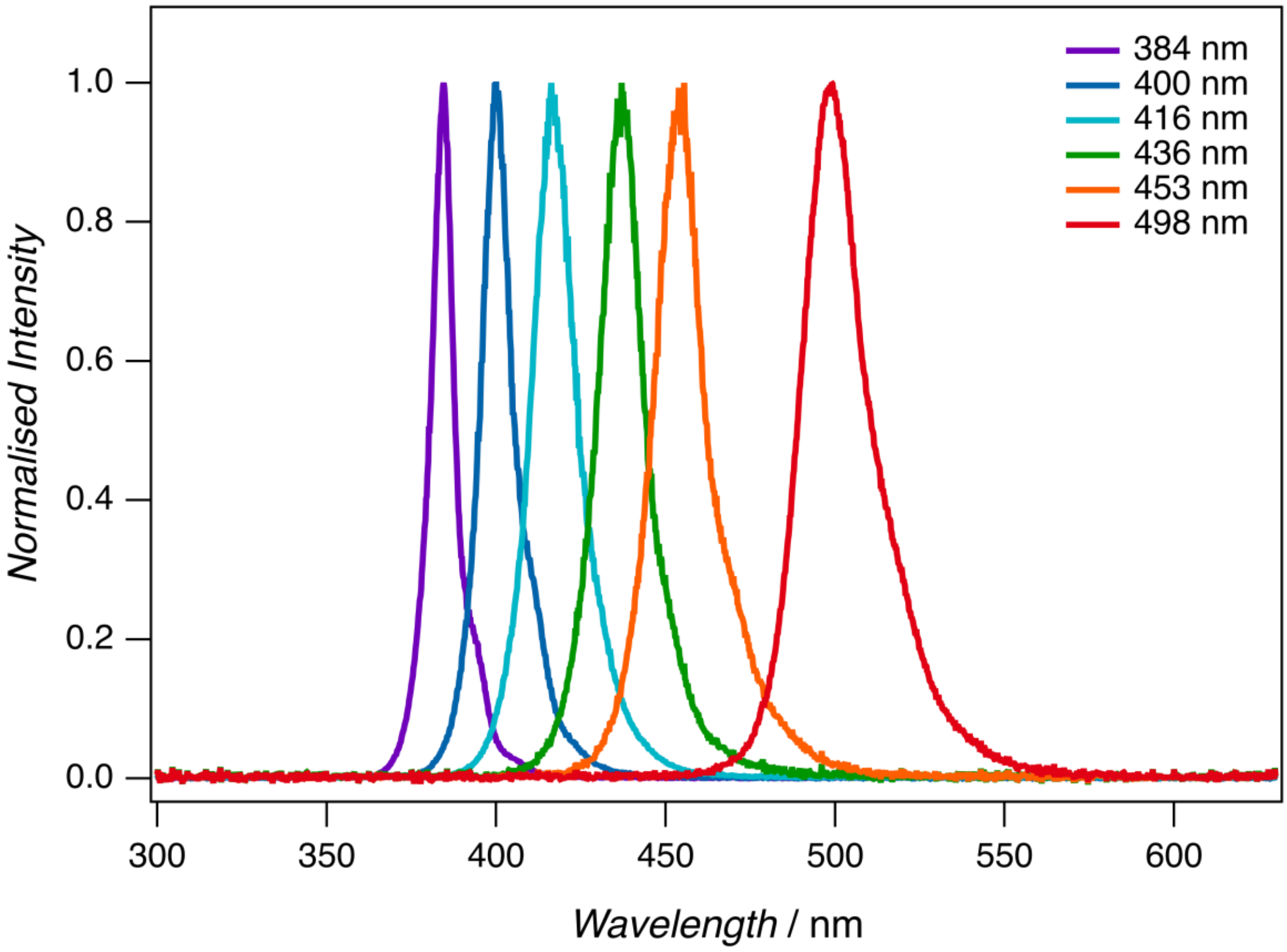

Figure S1. Emission spectra of LED light used for irradiation, i.e. λ_{max} = 360, 384, 400, 416, 436, 453, and 498 nm.

The peak emission position (λ_{max}) and full width – half maximum (*FWHM*) and their corresponding errors for each LED emission profile are shown in **Table S1** below. The errors on the λ_{max} position and *FWHM* were calculated from the location of the Gaussian maximum and calculation of the Gaussian *FWHM* during the fitting procedure.

Table S1. FWHM and standard deviation for each LED emission spectrum.

Nominal λ_{max} / nm	λ_{max} / nm	FWHM / nm
385	383.85 ± 0.03	7.49 ± 0.06
400	400.6 ± 1.4	13.4 ± 0.4
415	416.48 ± 0.03	13.35 ± 0.05
440	436.35 ± 0.04	14.21 ± 0.07
455	453.21 ± 0.04	14.49 ± 0.07
505	498.34 ± 0.04	16.56 ± 0.08

2 BAPO-NH_2 - Synthesis and Characterization

2.1 Synthesis

BAPO-NH_2 was prepared according to the synthesis reported by Thijssen *et al*. in 2024.[1]

2.2 Characterization via NMR

^{1}H NMR (400 MHz, $CDCl_3$): δ (ppm) = 6.87 (s, 4H), 3.31 (br, 2H), 2.30 (s, 12H), 2.29 (s, 6H).

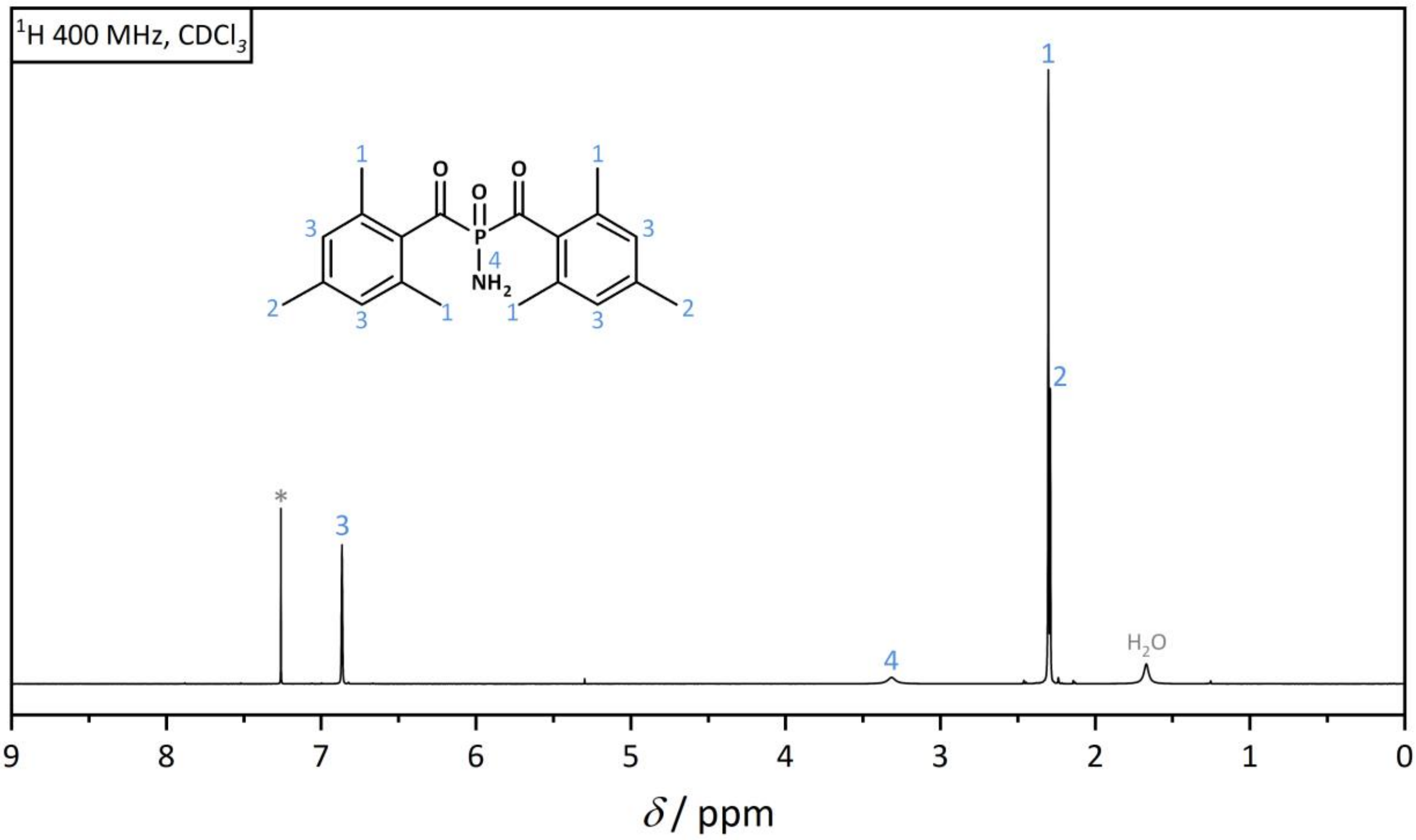

Figure S2. ^{1}H NMR (400 MHz) spectrum of BAPO-NH_2 in $CDCl_3$ (*) at ambient temperature.

^{13}**C NMR** (100 MHz, $CDCl_3$): δ (ppm) = 217.2, 216.4, 141.2, 135.9, 135.7, 135.5, 129.2, 21.4, 19.8.

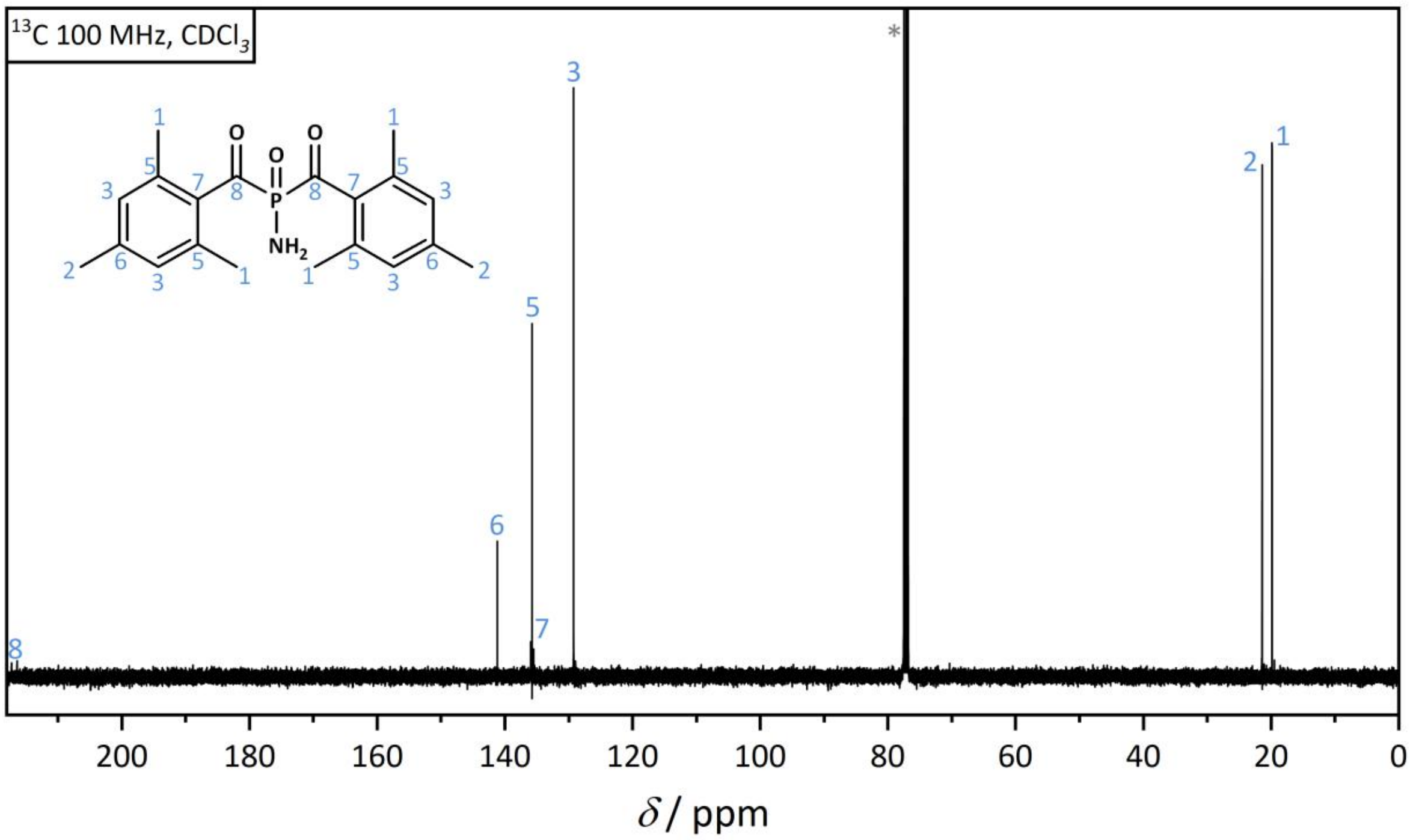

Figure S3. ^{13}C NMR (100 MHz) spectrum of BAPO-NH_2 in $CDCl_3$ (*) at ambient temperature.

^{31}**P NMR** (162 MHz, $CDCl_3$): δ (ppm) = 1.89.

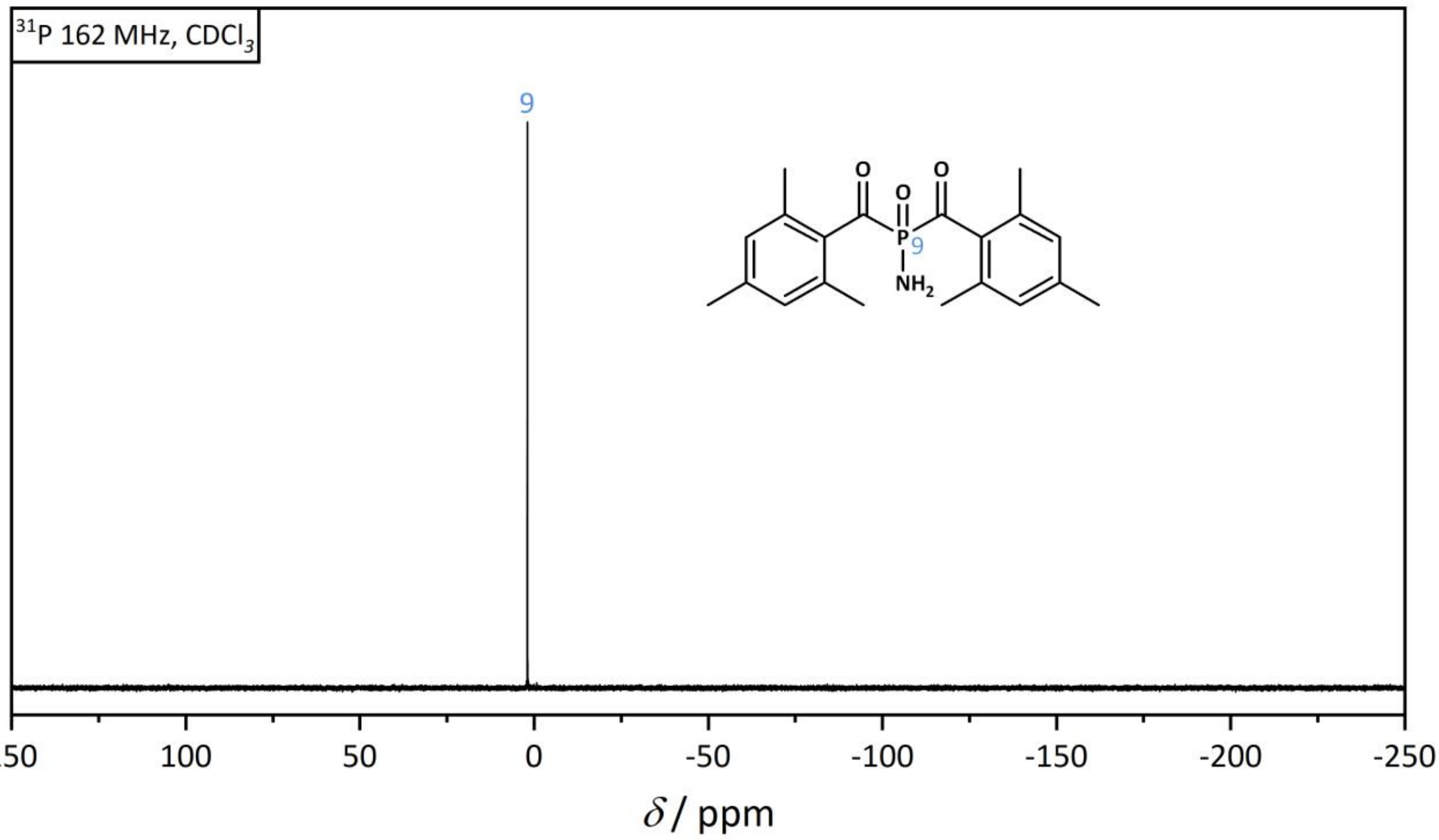

Figure S4. ^{31}P NMR (162 MHz) spectrum of BAPO-NH_2 in $CDCl_3$ at ambient temperature.

3 Preparation of the Photoresin

3.1 Preparation

The photoresin was prepared by mixing DMA (5.00 mL, 48.5 mmol) PEG-DA 700 (5.00 mL, 5.6 g), PEG 400 (50.0 mL, 56.4 g) and DMSO (40.0 mL, 43.8 g) in a flask at ambient temperature. BAPO-NH_2 (36.1 mg, 1.00 mmol) was dissolved in 20.2 mL of the mixture to prepare a 5 mM stock solution, which was subsequently used to prepare dilutions with different photoinitiator concentrations of the photoresin.

To determine a photoinitiator concentration that allowed complete acquisition of modulus-growth profiles without exceeding the measurable modulus range of the rheometer, kinetic screening experiments were performed using a structural variant of BAPO-NH_2, i.e. phenyl-bis(2,4,6-trimethylbenzoyl)phosphinoxide (BAPO) in the concentration range from 0.25 to 2.50 mM.

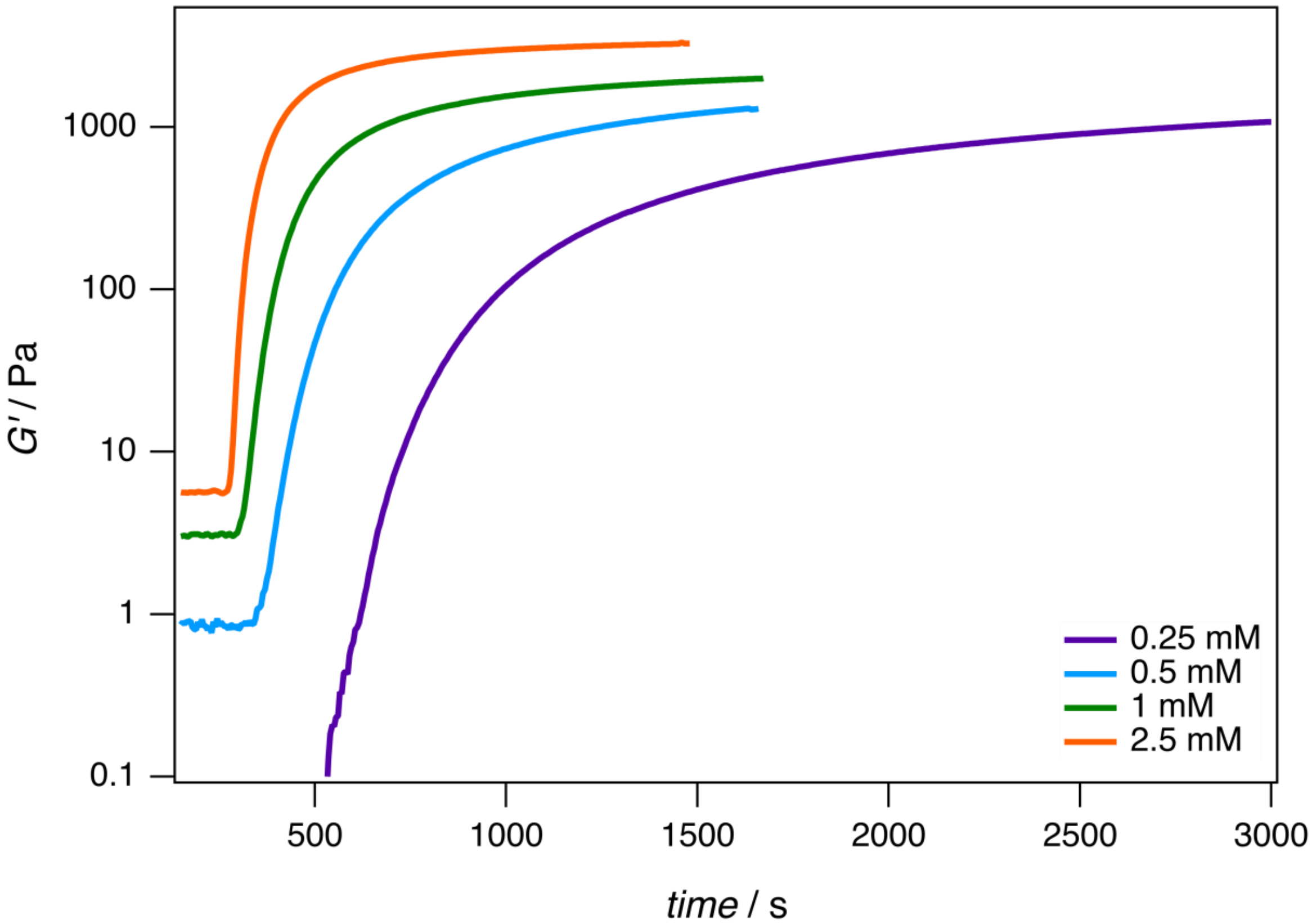

Figure S5. BAPO was used as the photoinitiator for all screening experiments as it is commercially available, given the more complex synthetic availability of BAPO-NH_2. Kinetic screening experiments were performed using λ_{max} = 410 nm, as it allowed for high conversion as observed by Thijssen *et al.*[1] Lower BAPO concentrations (0.25 - 0.50 mM) resulted in very

slow or incomplete curing, whereas higher concentrations (2.50 mM) produced rapid gelation that limited data collection due to the instrument's data acquisition rate. A concentration of 1 mM of photoinitiator was therefore selected for all wavelength-dependent experiments. The kinetics screening test was repeated with very similar results on a single concentration of 1 mM BAPO-NH_2 to ensure that the selection was valid for both photoinitiator moieties.

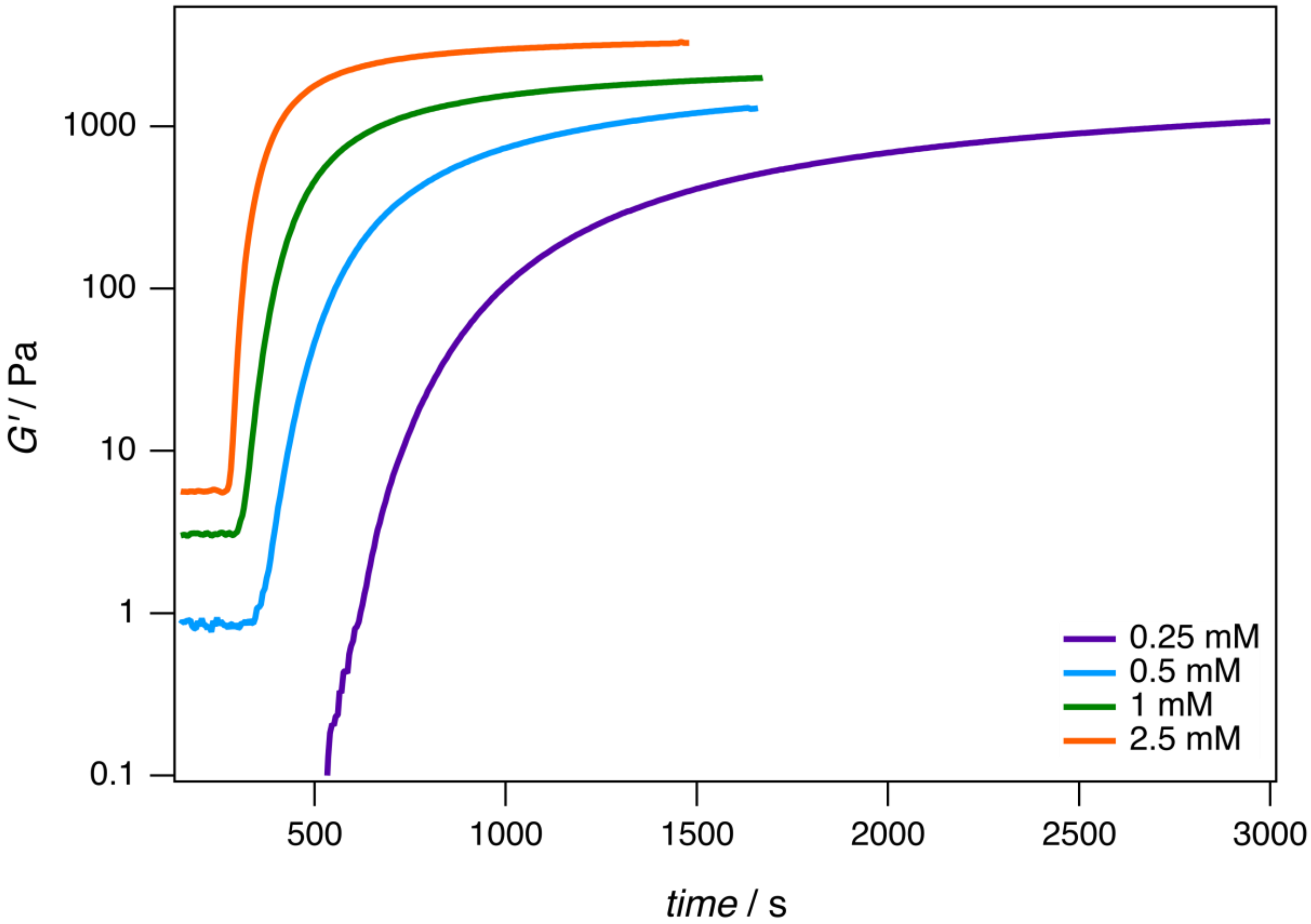

Figure S5. Kinetic experiments performed at λ_{max} = 410 nm for the curing of the photoresin with different concentrations of BAPO, averaged from three runs per concentration. The evolution of the storage modulus is plotted as a function of time, so that an appropriate concentration can be selected where the sample cures neither to rapidly nor too slowly.

3.2 UV-Vis Absorbance of the Photoresin

The UV-Vis absorbance spectra of BAPO-NH_2 were recorded at 1 mM concentration in both the MMA and in the curing resin (**Figure S6**) to enable a better comparison of the rheological and the gravimetric action plot recorded earlier in dilute solution.[1] Both spectra exhibit highly similar overall band shapes, indicating comparable absorbance behavior in both environments. Consequently, the action plots can be directly compared and differences mainly arise due to the method of data acquisition, which supports using an approach based on system-specific action plot measurements.

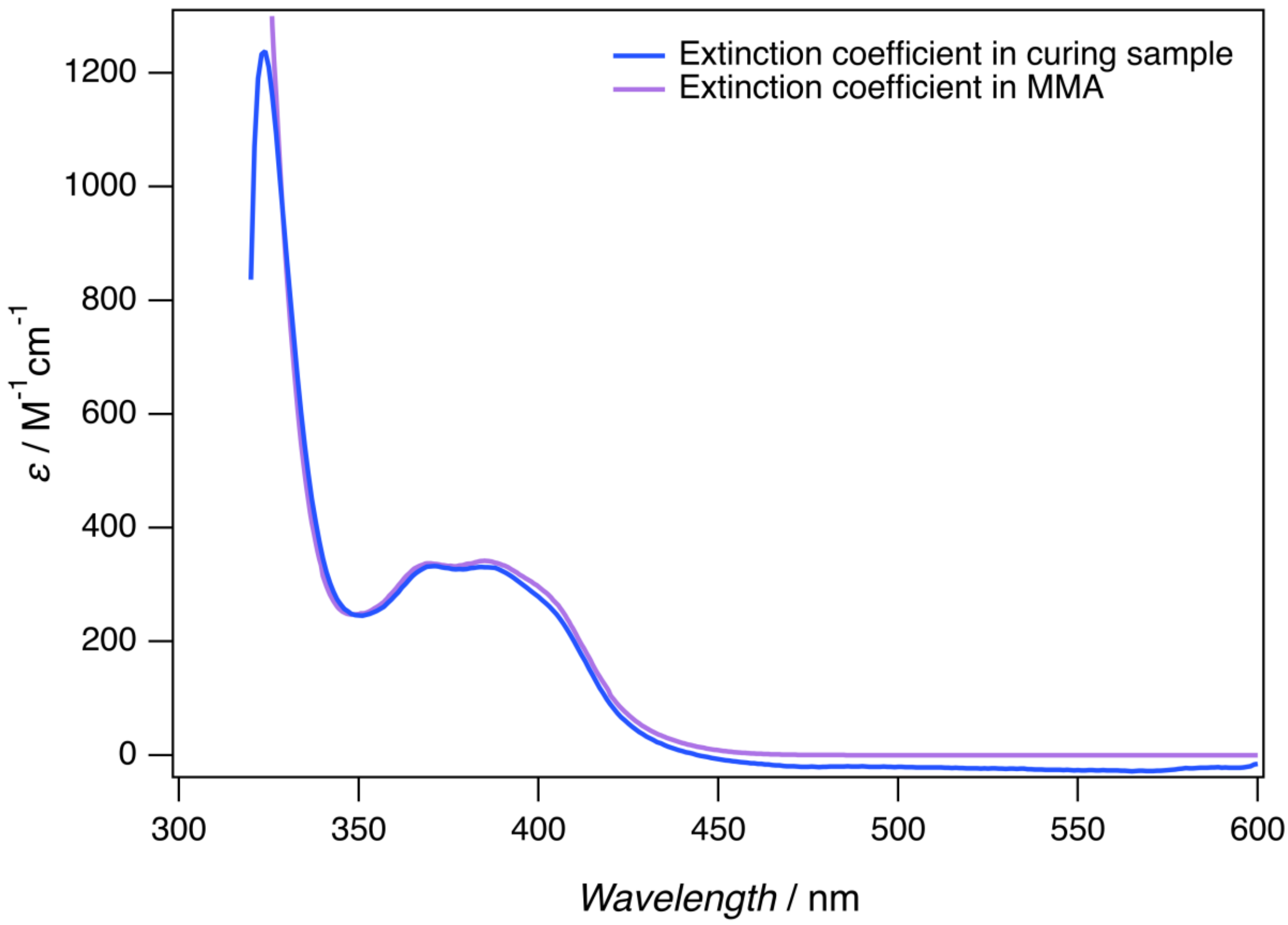

Figure S6. UV-Vis absorbance spectra of 1 mM concentration of BAPO-NH_2 in MMA[1] and in the herein employed curing formulation.

3.3 Transmittance of the Photoresin

Transmittance was calculated from UV-Vis absorbance data using Beer-Lambert's law, evaluated for sample thicknesses between 0.05 mm and 0.8 mm (corresponding to typical rheometer gaps) as shown in **Figure S7**. Transmittance exceeded 95 % for wavelengths above approximately 340 nm across all thicknesses, confirming that optical attenuation is negligible in the spectral region used for photorheological measurements.

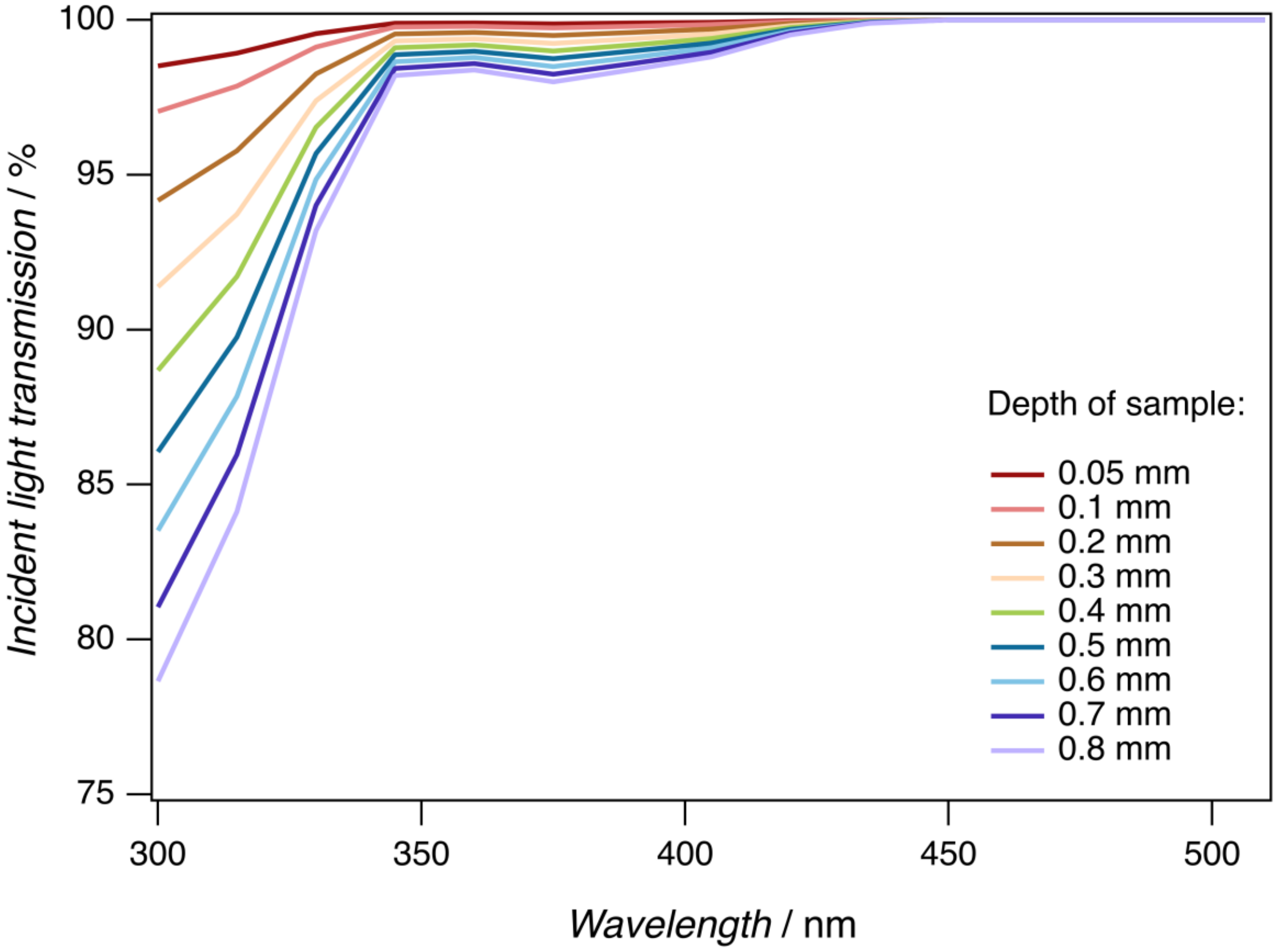

Figure S7. Calculated transmittance of incident light through the photoresin with 1 mM BAPO-NH_2 as a function of wavelength for different sample thicknesses.

3.4 Light Absorbance in the Photoresin

Absorbance in the photoresin sample was assessed as a qualitative assessment of scattering by loading the sample into a 22 mm quartz cuvette and exposing it to a 10W 410 nm LED at 9.1W electrical power at a distance of 1.5 cm from the cuvette. The sample was irradiated for 0, 60, 300 and 600 s and the absorbance measured after each exposure (refer to **Figure S8**). A decrease in the MesBAPO-NH_2 absorbance band and an increasing extinction tail up to 700 nm is observed. Since none of the components have a meaningful extinction above 500 nm, the slight increase in the extinction tail may represent a small amount of scattering as the sample cures. The assessed sample is 10 times thicker than the rheology sample. The rheology sample was optically transparent at all times during the measurement and since the amount of scattering recorded in the much optically thicker sample appears to be very minor, scattering was not considered a significant factor in the photorheology experiments.

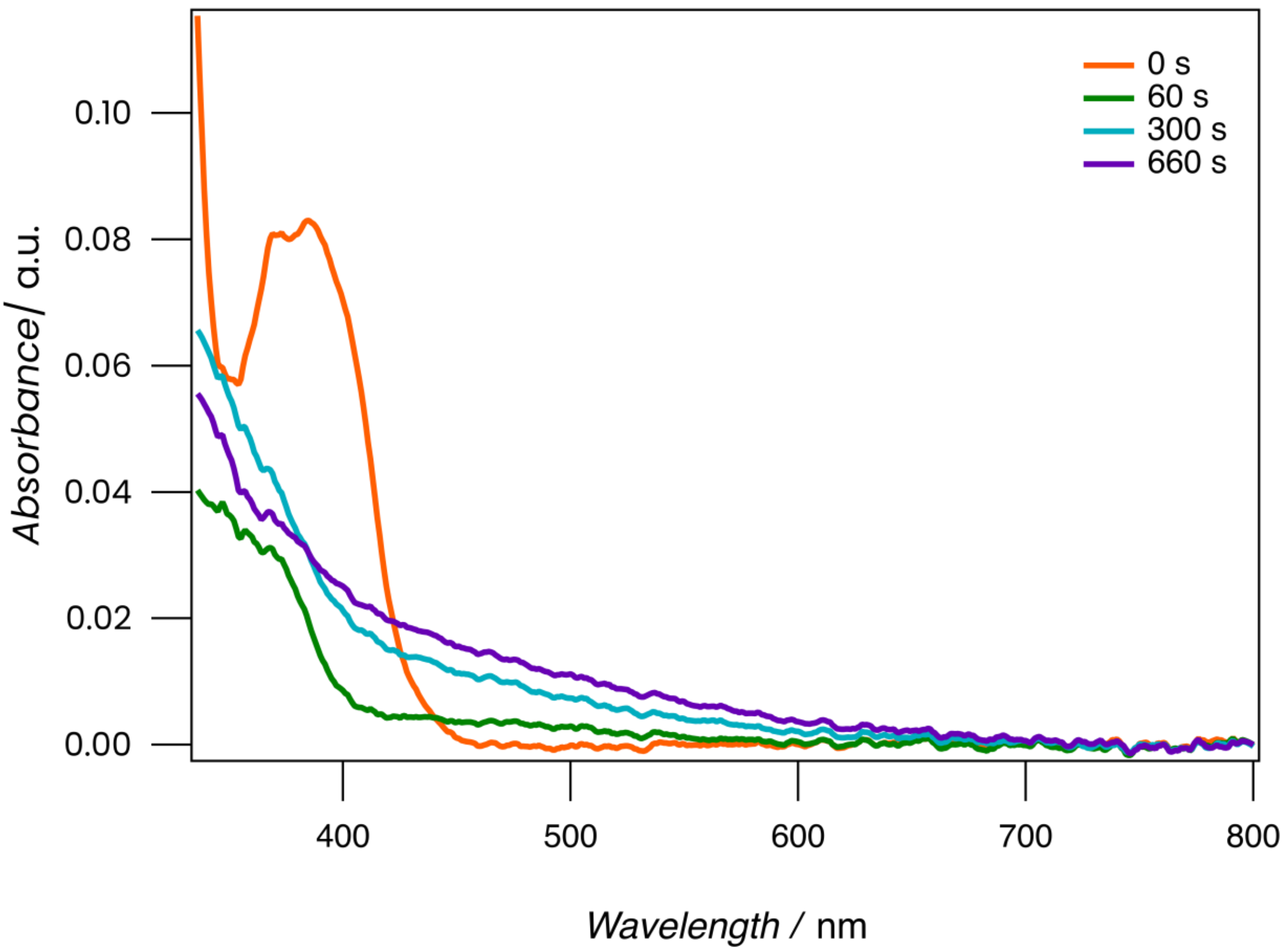

Figure S8. Absorbance of the photoresin used in the photorheology experiments after exposure to irradiation at λ_{max} = 410 nm for 60, 300 and 660 s. The experiment shows that as the chromophore was consumed, there was a slight rise in the absorbance above 500 nm, probably due to a small amount of scattering in the sample.

4 Photorheological Measurements

4.1 Photorheometer Setup

A custom optical alignment apparatus was developed to ensure that the incident light was vertically collimated and centered precisely at the midpoint of the rheological sample of an *Anton Paar* MCR302 rheometer from below. A quartz lower plate was incorporated into the rheometer geometry to maximize transmission across the employed wavelength range and to prevent attenuation artefacts associated with borosilicate glass plates.

The alignment system consisted of a positioning plate and spacer assembly with a threaded aperture at the top of the spacer used to secure the collimating lens (*Thorlabs* LA1951-ML). The positioning plate contained a recessed circular region matching the diameter of the LED heat-sink housings, ensuring that each LED was reproducibly located beneath the center of the measuring plate. The plate assembly was anchored to the rheometer using threaded rods that interfaced with pre-installed mounting fixtures, providing consistent spatial registration across all experiments. The measuring-plate height was adjusted using a threaded riser, allowing fine control of the photon flux delivered to the sample by varying the optical path length. A schematic and photograph of the complete assembly are shown in **Figure S9**.

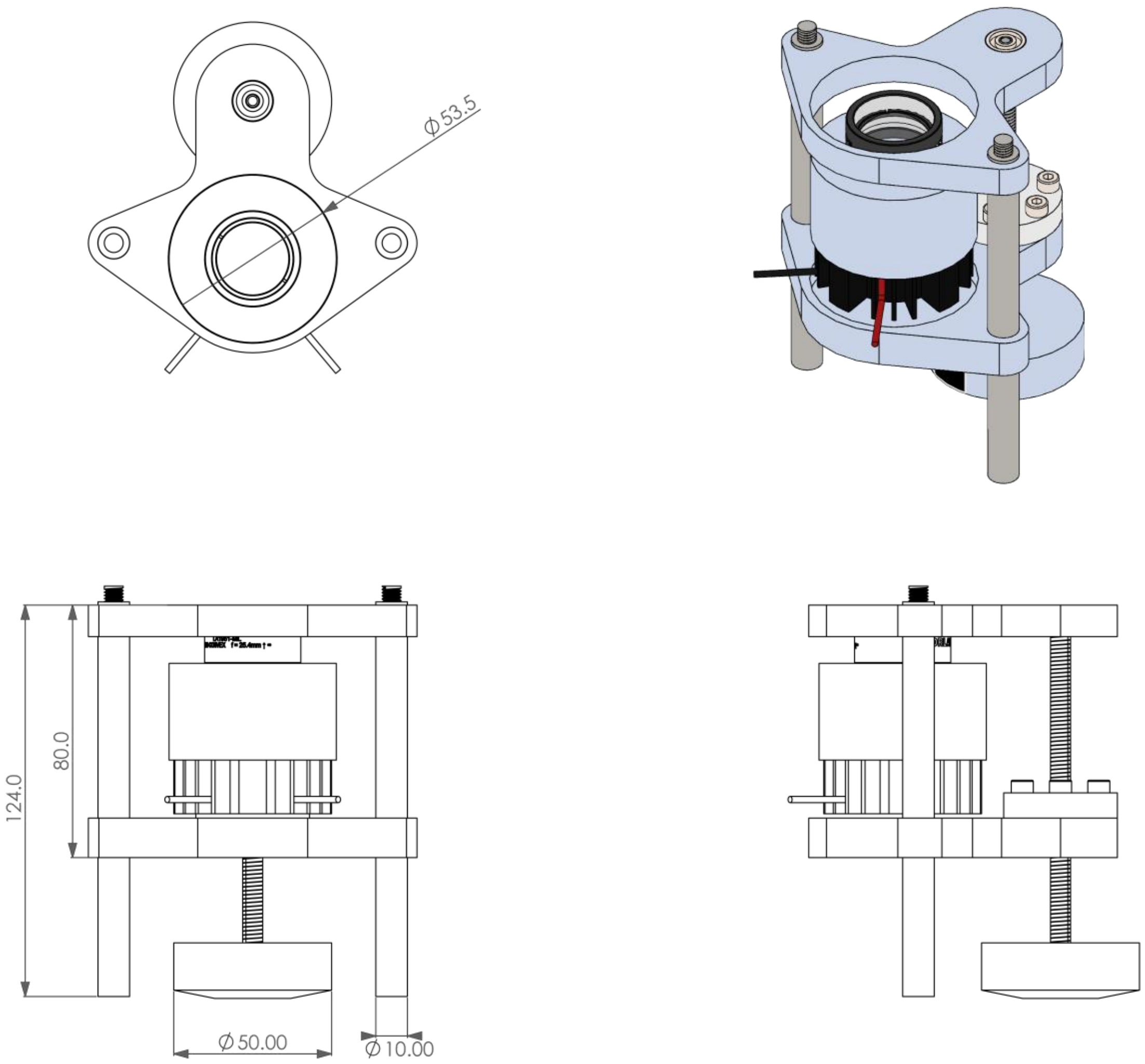

Figure S9. Custom-designed optical alignment apparatus for controlled irradiation during photo-rheological measurements. (Top) CAD rendering of the positioning plate and spacer assembly, featuring a recessed region for locating LED heat-sink housings and a threaded aperture for mounting the collimating lens. (Bottom) Side views of the full assembly, showing the adjustable threaded riser used to set the LED to sample distance and the alignment rods that register the system to the rheometer. The device ensures vertically collimated light passing through the quartz lower plate and precise, reproducible central alignment of the LED with the sample position.

All LEDs were operated using a *Tenma* 72-2685 30V 3A digital control DC variable power supply and their output intensities were adapted to the maximum stable photon flux of the weakest LED (approximately $5 \cdot 10^{15}$ photons s^{-1}) to maintain comparable irradiation conditions across wavelengths. Irradiance measurements were conducted using a calibrated *Thorlabs* PM400 optical power meter equipped with a S401C thermal power sensor. To ensure precise and reproducible positioning of the power meter, a custom aluminum holder was machined with

an internal thread matching the power-meter cap. The holder was designed to slide directly over the rheometer measuring plate, positioning the detector at the exact location occupied by the sample and at the geometric center of the plate (**Figure S10**). This configuration enabled wavelength-independent irradiance measurements and ensured that all subsequent curing experiments were performed under well-defined and fully repeatable optical conditions. The average calculated photon flux was $5{\cdot}10^{15}$ photons s^{-1}. **Figure S11** shows a selection of photographs illustrating the LED and lens assembly, the locating plate for power assessment and the positioning of the device below the rheometer.

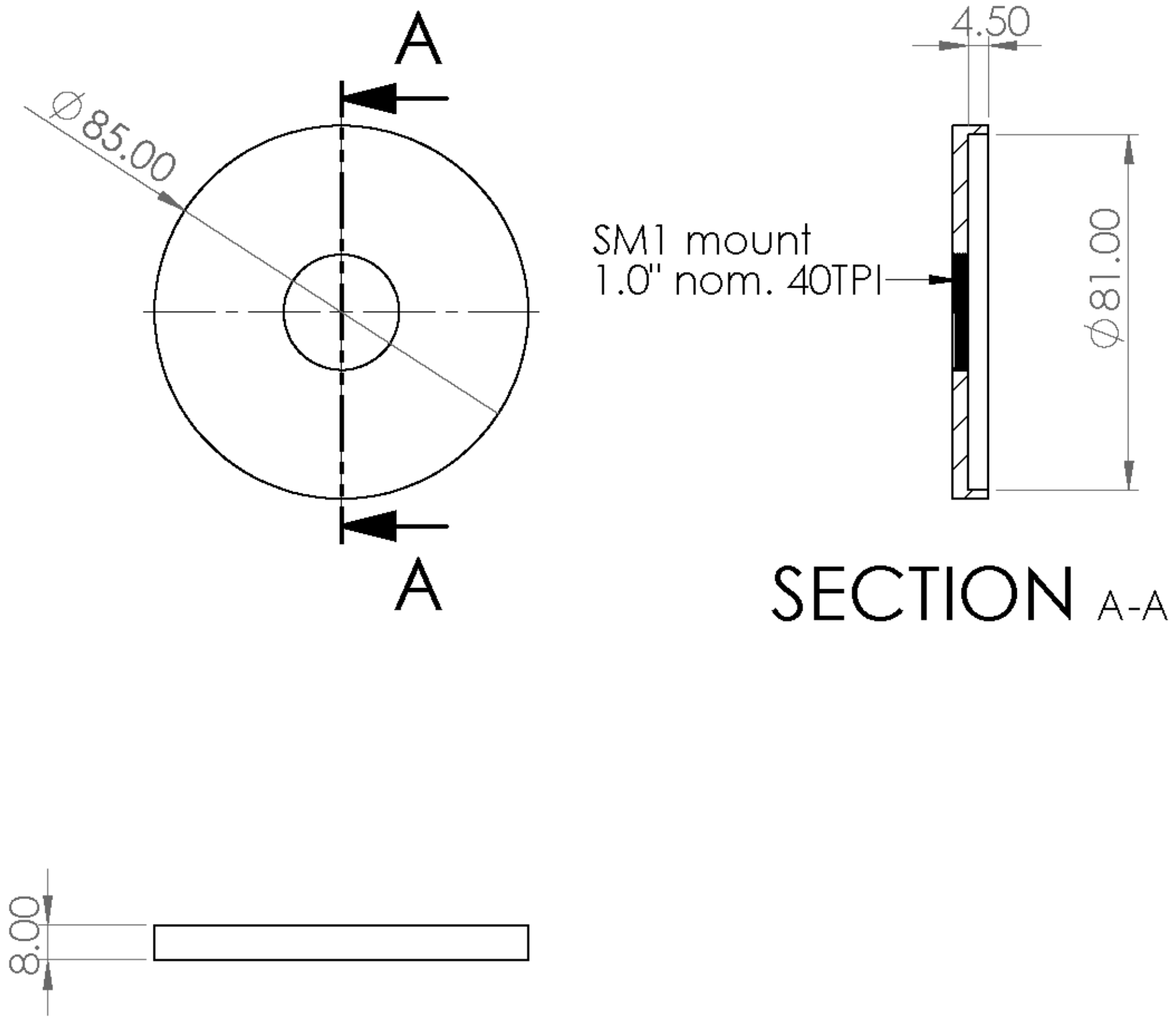

Figure S10. Custom-designed alignment apparatus to ensure accurate and reproducible placement of the optical power meter for measurement of LED power at each selected wavelength. This assembly is separate to the above device (Figure S9) and sits on top of the rheometer plate when measuring the LED power outputs. (Top left) CAD rendering of the positioning plate assembly, featuring a threaded region for locating the optical power sensor. (Top right) Section through assembly center showing details of thread required. (Bottom) Side view of the plate assembly. The device ensures reproducible central alignment of the optical power meter above the center of the rheometer's measuring plate.

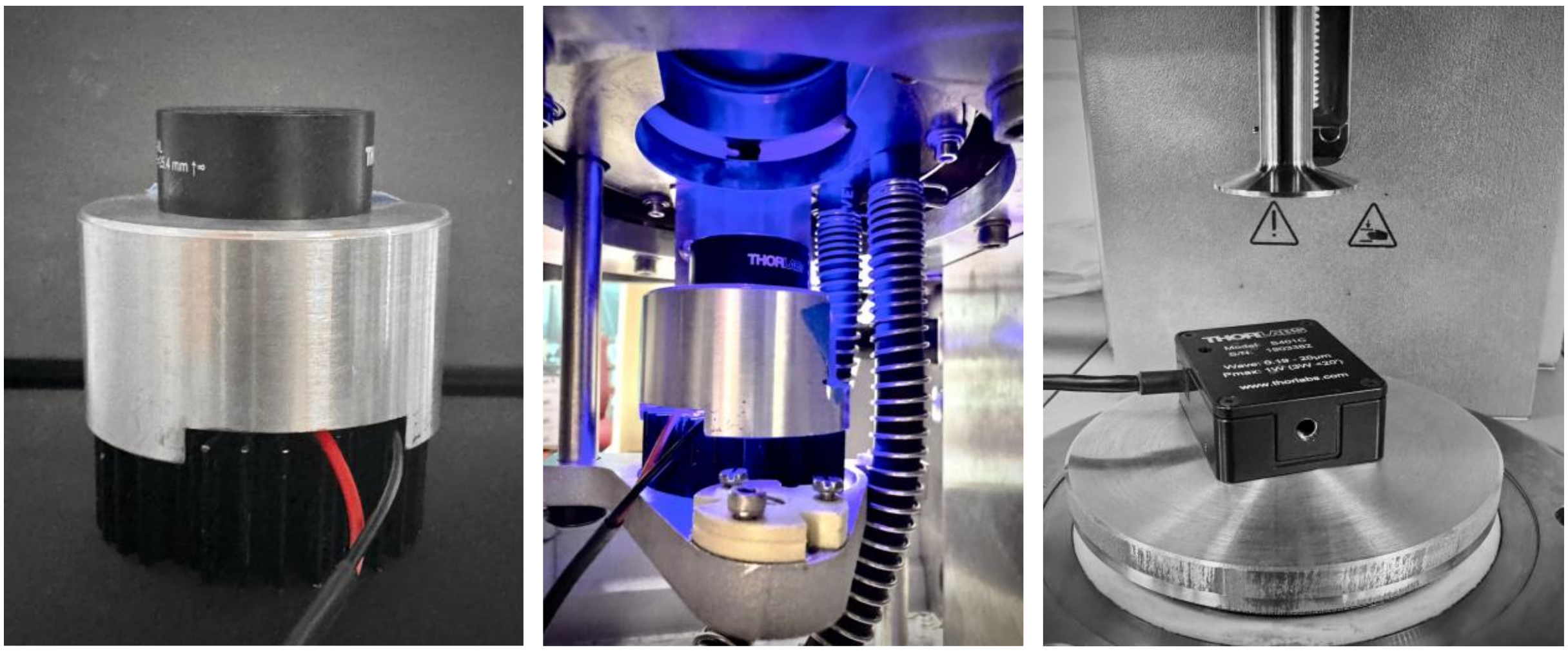

Figure S11. Photographs of the lens assembly, power meter location assembly and LED assembly set in place below the rheometer plate. (Left) LED mounted on cooling device with an aluminum spacer and collimating lens installed. (Centre) Fully assembled LED positioning device, placed below the rheometer plate and irradiating the curing sample. (Right) Power meter sensor mounted onto locating device and placed on the rheometer plate, directly above the LED and lens.

Rheological measurements were performed on an *Anton Paar* MCR302 rheometer equipped with a quartz lower plate and a 25 mm parallel-plate geometry to ensure uniform sample thickness. All experiments were conducted at 25 °C with temperature controlled by the instrument's Peltier system. Frequency and amplitude sweeps recording storage modulus (*G'*) and loss modulus (*G''*) were carried out on the photoresin before curing and fully cured (respectively) prior to irradiation experiments to establish suitable linear-viscoelastic operating conditions (refer to **Figure S12** and **Figure S13**). Based on these preliminary tests and the optical transmission calculations shown in **Figure S7**, an oscillatory strain of 0.5 %, a frequency of 5 Hz, and a measurement gap of 0.20 mm were selected for all photo-rheological measurements. Such experiments typically use 1 Hz, but the frequency loss modulus data was very noisy still at 1 Hz, suggesting that the torque measured was lower than the detection limit of the instrument. The amplitude sweep shows that the linear viscoelastic range (LVER) extends to a strain value of around 0.5 (noting that the axes are logarithmic and therefore the slight decrease in loss modulus is amplified here).

The measurement gap was set to 0.2 mm at the start of the experiment and the instrument was operated in normal force control mode. The normal force and gap were recorded at each data point throughout every experiment and checked to ensure that the sample did not show signs of excessive shrinkage or expansion during the experiment. The recorded gap ranged between 0.197 – 0.206 mm and the normal force showed a variation of < 0.5 N over all experiments.

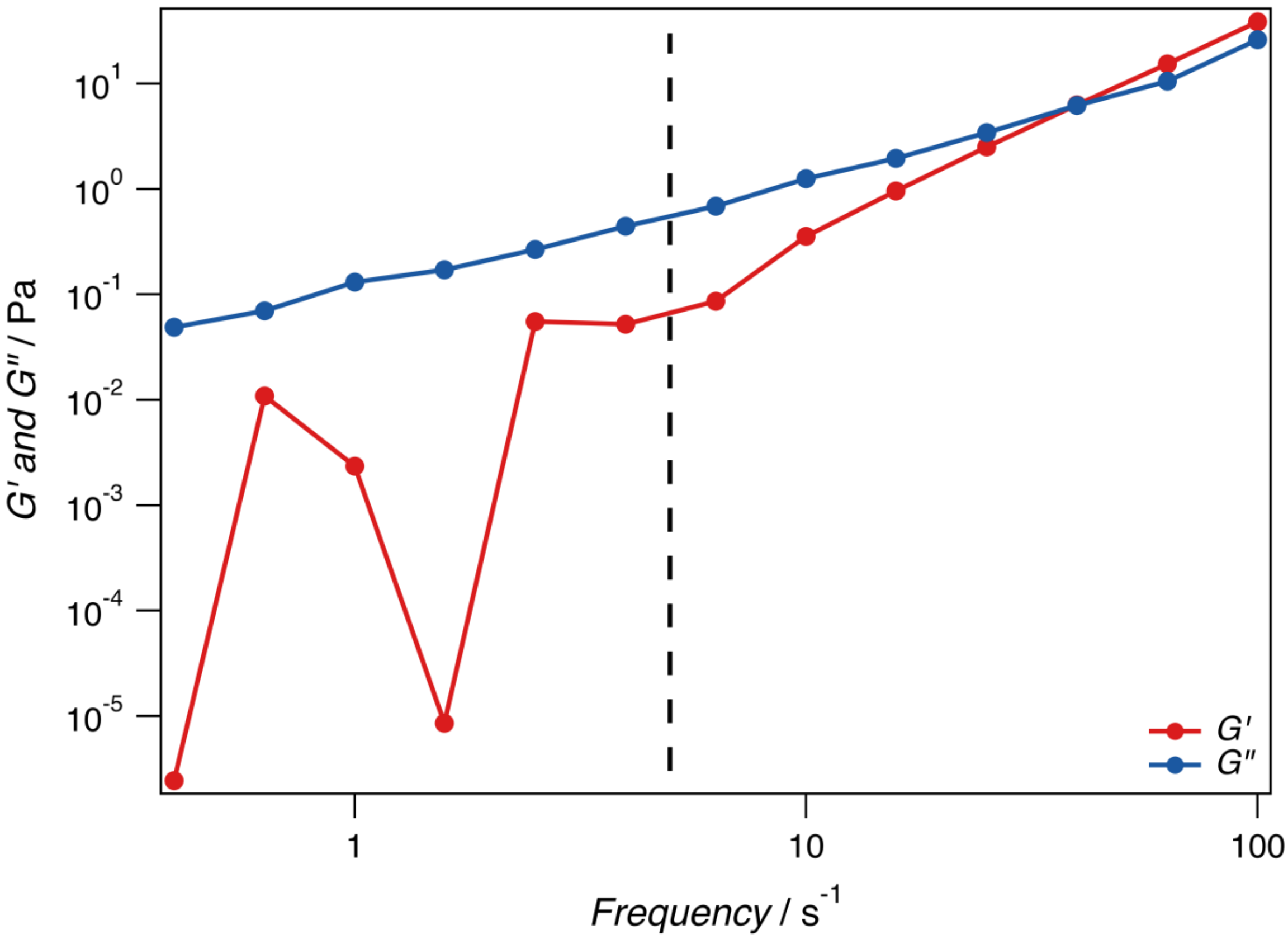

Figure S12. Pre-cure frequency sweep of the photoresin based on DMA, PEG-DA 700, DMSO and PEG 400 at volumetric ratios of 5% (486 mM) 5% (0.08 M), 40% (5.63 M), 50% (1.4 M), respectively and BAPO (1 mM) as photoinitiator. The dashed line shows the frequency selected for the tests, where the torque is high enough to give reliable data.

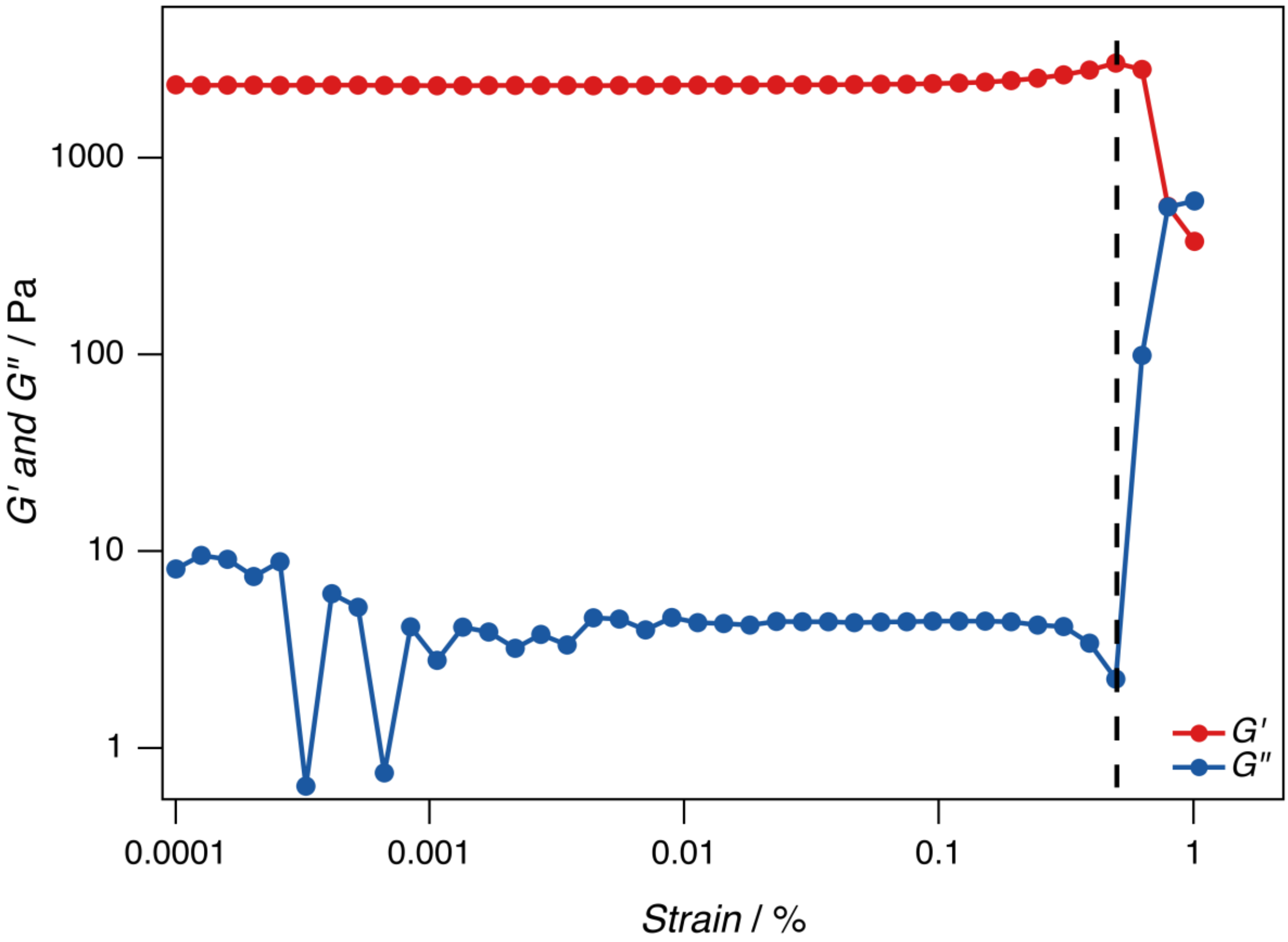

Figure S13. Amplitude sweep performed on the cured photoresin based on DMA, PEG-DA 700, DMSO and PEG 400 at volumetric ratios of 5% (486 mM) 5% (0.08 M), 40% (5.63 M), 50% (1.4 M), respectively and BAPO (1 mM) as photoinitiator. The dashed line shows the selected oscillation amplitude, at the highest point of the linear viscoelastic range (LVER). Note that the y-axis is logarithmic, thus the decrease in loss modulus at this point is relatively minor compared to the storage modulus value. The maximum strain within the LVER is chosen to give the best chance at obtaining good data from the sample before curing.

4.2 Photorheological Experiments

For each selected wavelength, the evolution of the storage modulus (*G'*) of the photoresin was recorded as a function of irradiation time and thus photon number. The temporal axis was converted to a photon-dose axis using irradiance values. Each measurement was performed in triplicate, with irradiance recorded immediately before and after each run. The two values were averaged to account for small fluctuations in LED output and the averaged irradiance was used to calculate the total photon dose delivered during the evolution of *G'*.

5 Construction of Photorheological Action Plots for BAPO-NH_2

5.1 Different Types of Photorheological Action Plots

The evolution of G' during the curing of a photoresin at different wavelengths offers several approaches for evaluating the data in the context of a photochemical action plot. We propose four different methods: Fixed photon dose, maximum rate of change of G', onset of curing and number of photons to the maximum rate of change of G' (**Figure S14**).

Figure S14 shows two examples of the derivation of these curves. In **Figure S14 a**, the G'-N curves are shown with an intersecting line at $N = 2{\cdot}10^{18}$. When each of the G'- curves is analysed in this fashion, the G' values at the intersection point with the fixed N value can be plotted vs. the wavelength alongside the molar extinction curve as shown in **Figure S14 c**. The second approach shown entails finding the maximum rates of curing ($\mathrm{d}G'/\mathrm{d}N_{\mathrm{max}}$), determined by differentiating G' with respect to photon dose and identifying the peak value of the differentiated curve. The differentiation was performed on G' curves averaged from three experiments and the differentiated curve smoothed using a Savitzky-Golay algorithm with a window size of 5 points. There is a clear and unambiguous maximum in each of these curves and the method is free from subjective analysis. This method is also independent of arbitrary choice of N, allowing for a more wholistic interpretation of the data. The differentiated curves are shown in **Figure S14 b**. When each value of dG'/dN_{max} is plotted against its corresponding wavelength, the action plot shown in **Figure S14 d** is derived. This plot also shows a red shift of close to 15 nm from the first local maximum of the absorbance spectrum at 385 nm to that of the action plot at 400 nm. A comparison of the action plots derived using these methods is shown in **Figure S15 a**.

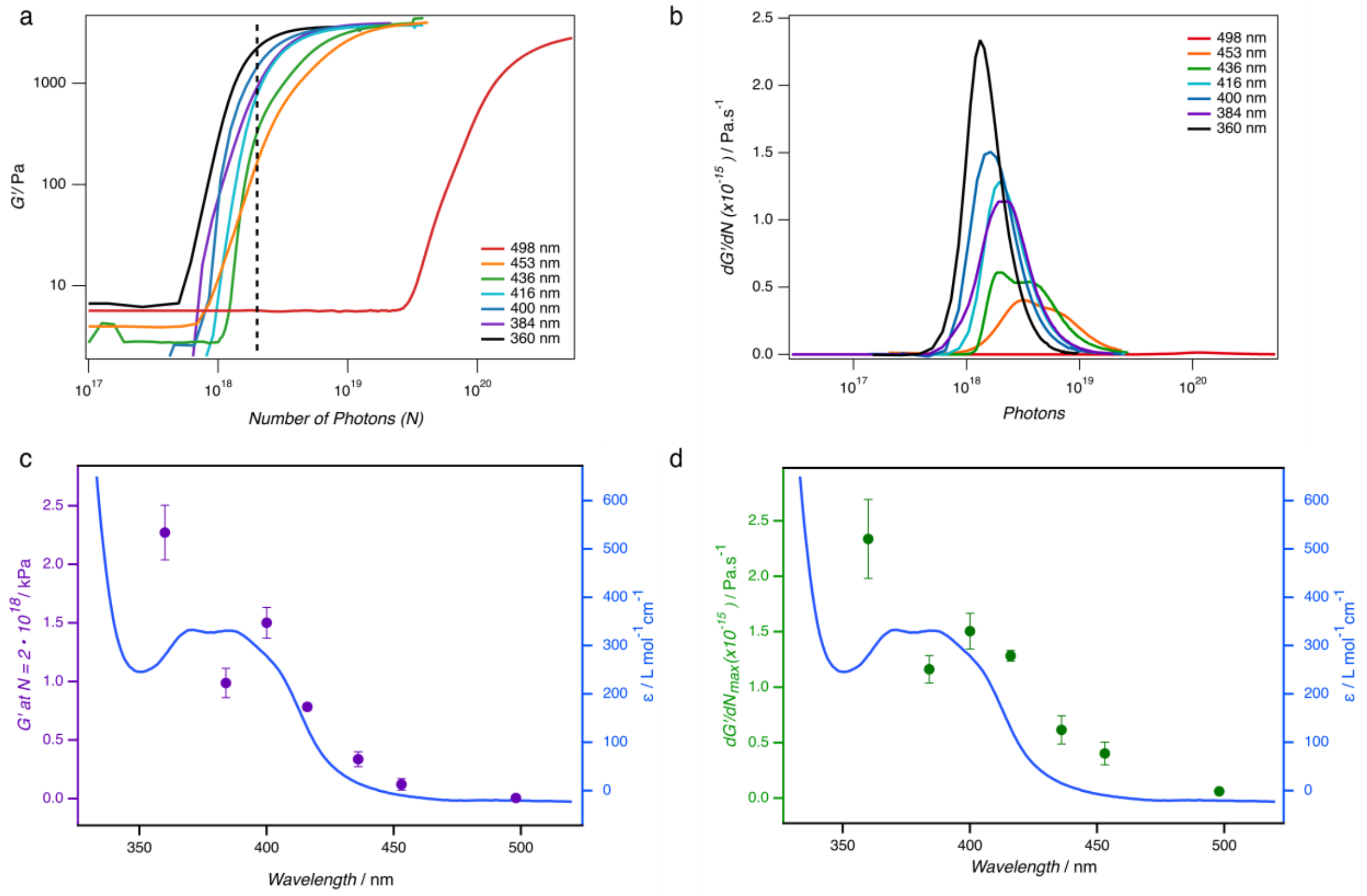

Figure S14. Comparison of different approaches to construct an action plot. **a** All *G'* curves measured under irradiation at λ_{max} = 360 - 498 nm with a dotted line marking *G'* at $2 \cdot 10^{18}$ photons exposure. **b** All derivatives of *G'*, measured under irradiation to full curing at λ_{max} = 360 - 498 nm. **c** Fixed dose rheological action plot depicting *G'* for different wavelengths at a photon number of $2 \cdot 10^{18}$. **d** Maximum rate of change of *G'* rheological action plot depicting the maximum rates of change of *G'* for different wavelengths.

5.2 Alternative Methods for Photorheology Action Plot Construction

The photon dose at the onset of curing was defined as the point where the storage modulus increased beyond 10% of its pre-cure value (to ensure that the increase was above the signal to noise ratio) and continued to increase. The 10% increase was chosen rather than the 3% threshold that is common in rheology,[2] because as the sample is relatively liquid at the start, *G'* is low and the instrument torque values are contributing to the errors. The number of photons to the maximum rate of curing (*dG'/dN)* was evaluated as another possible data analysis method. The values for the number of photons at onset (N_{on}) and number of photons to reach *dG'/dN* (N_{max}) were both reciprocated to reflect the fact that if a lower number of photons is required to reach the curing onset and the maximum rate of reaction, the reaction is proceeding more efficiently at a given wavelength. **Table S2** shows the reciprocal values of the number of photons to the onset of curing and to the maximum rate of curing for each LED wavelength and **Figure S15 b** shows the action plots derived using these methods.

Table S2. Reciprocal values of N to reach the onset of curing ($1/N_{on}$) and the maximum rate of curing ($1/N_{max}$) for each LED wavelength.

λ_{max} (nm)	$1/N_{on} \cdot 10^{-19}$	$1/N_{max} \cdot 10^{-19}$
383.85 ± 0.03	20.16 ± 0.11	7.60 ± 0.04
400.6 ± 1.4	16.42 ± 0.10	5.22 ± 0.03
416.48 ± 0.03	15.26 ± 0.10	6.13 ± 0.04
436.35 ± 0.04	13.63 ± 0.09	5.13 ± 0.03
453.21 ± 0.04	9.91 ± 0.07	5.12 ± 0.04
498.34 ± 0.04	14.54 ± 0.35	3.18 ± 0.08
383.85 ± 0.03	0.44 ± 0.00	0.09 ± 0.00

5.3 Visualization of Photorheological Action Plot Evaluation Methods

The two most promising methods for construction a rheological action plot were the fixed number of photons method and the dG'/dN_{max} method (**Figure S15 a**). The data from the number of photons to the start of curing and the dG'/dN_{max} were less well resolved (**Figure S15 b**).

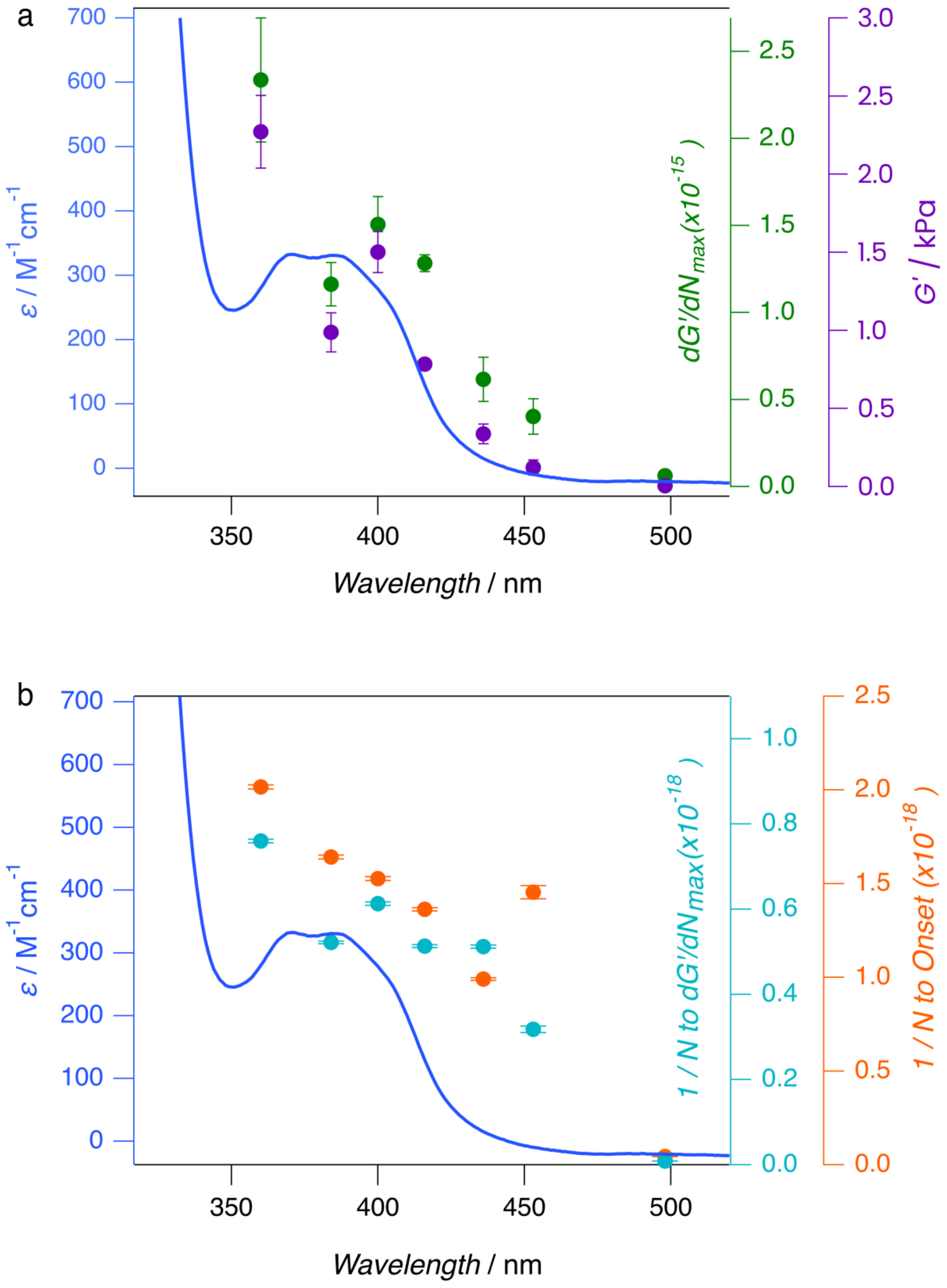

Figure S15. a Fixed dose rheological action plot depicting *G'* for different wavelengths at a photon number of $2 \cdot 10^{18}$ and dG'/dN_{max} rheological action plot depicting the maximum rates of change of *G'* for different wavelengths. **b** Action plots derived from assessing the number of photons to reach the reaction onset and dG'/dN_{max}. The action plots shown at the top figure appear to be better resolved than those shown in the bottom figure.

6 References

1 Q. Thijssen, J. A. Carroll, F. Feist, A. Beil, H. Grützmacher, M. Wegener, S. Van Vlierberghe and C. Barner-Kowollik, *Materials Horizons*, 2024, **11**, 6184-6191.

2 T. G. Mezger, *Applied rheology: With Joe Flow on rheology road*, Anton Paar GmbH, Austria, 2014.
